# Nanomedicine Targeting Cuproplasia in Cancer: Labile Copper Sequestration Using Polydopamine Particles Blocks Tumor Growth *In Vivo* through Altering Metabolism and Redox Homeostasis

**DOI:** 10.1021/acsami.4c04336

**Published:** 2024-06-03

**Authors:** Javier Bonet-Aleta, Miguel Encinas-Gimenez, Miku Oi, Aidan T. Pezacki, Victor Sebastian, Alba de Martino, Ana Martín-Pardillos, Pilar Martin-Duque, Jose L. Hueso, Christopher J. Chang, Jesus Santamaria

**Affiliations:** † Instituto de Nanociencia y Materiales de Aragon (INMA) CSIC, Universidad de Zaragoza, Campus Rio Ebro, Edificio I+D, C/Poeta Mariano Esquillor, s/n, 50018 Zaragoza, Spain; ‡ Networking Res. Center in Biomaterials, Bioengineering and Nanomedicine (CIBER-BBN), Instituto de Salud Carlos III, 28029 Madrid, Spain; § Department of Chemical and Environmental Engineering, University of Zaragoza, Campus Rio Ebro, C/María de Luna, 3, 50018 Zaragoza, Spain; ∥ Department of Chemistry, University of California, Berkeley, California 94720, United States; ⊥ Instituto Aragonés de Ciencias de la Salud (IACS), Instituto de Investigación Sanitaria Aragón (IIS-Aragón), Edificio CIBA. Avenida San Juan Bosco 13, planta 1, 50009 Zaragoza, Spain; ∇ Department of Molecular and Cell Biology, University of California, Berkeley, California 94720, United States; ○ Helen Willis Neuroscience Institute, University of California, Berkeley, California 94720, United States; ● Instituto de Investigación Sanitaria (IIS) de Aragón, Avenida San Juan Bosco, 13, 50009 Zaragoza, Spain; ▲ Departamento de Desarrollo de Medicamentos y Terapias Avanzadas, Instituto de Salud Carlos III, Ctra. de Pozuelo, 28, 28222, Majadahonda Madrid, Spain

**Keywords:** copper, polydopamine, labile copper, bioimaging, cancer metabolism

## Abstract

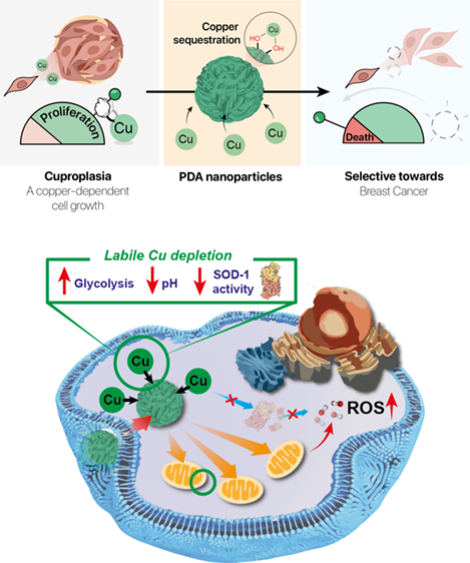

Copper plays critical roles as a metal active site cofactor and metalloallosteric signal for enzymes involved in cell proliferation and metabolism, making it an attractive target for cancer therapy. In this study, we investigated the efficacy of polydopamine nanoparticles (PDA NPs), classically applied for metal removal from water, as a therapeutic strategy for depleting intracellular labile copper pools in triple-negative breast cancer models through the metal-chelating groups present on the PDA surface. By using the activity-based sensing probe FCP-1, we could track the PDA-induced labile copper depletion while leaving total copper levels unchanged and link it to the selective MDA-MB-231 cell death. Further mechanistic investigations revealed that PDA NPs increased reactive oxygen species (ROS) levels, potentially through the inactivation of superoxide dismutase 1 (SOD1), a copper-dependent antioxidant enzyme. Additionally, PDA NPs were found to interact with the mitochondrial membrane, resulting in an increase in the mitochondrial membrane potential, which may contribute to enhanced ROS production. We employed an *in vivo* tumor model to validate the therapeutic efficacy of PDA NPs. Remarkably, in the absence of any additional treatment, the presence of PDA NPs alone led to a significant reduction in tumor volume by a factor of 1.66 after 22 days of tumor growth. Our findings highlight the potential of PDA NPs as a promising therapeutic approach for selectively targeting cancer by modulating copper levels and inducing oxidative stress, leading to tumor growth inhibition as shown in these triple-negative breast cancer models.

## 1 Introduction

Copper is subjected to delicate balance in living systems. This metal nutrient is required as an active site metabolic cofactor or metalloallosteric signaling agent in proteins to sustain key biological processes, yet copper excess is associated with potential oxidative stress and cytotoxicity.^1,2^ Indeed, impairment in intracellular copper levels is also associated with several tissue abnormalities and diseases.^3^ Emerging studies link the aberrant hyperaccumulation of copper levels in different cancers including breast,^4,5^ lung,^6^ or prostate^7^ with their growth and proliferation, grouped in a new concept termed “cuproplasia”.^8^ For instance, copper plays a pivotal role in energy metabolism as a cofactor of cytochrome *c* oxidase, an enzyme present in the inner mitochondrial membrane involved in ATP biosynthesis. It has been established that removing mitochondrial copper is an effective strategy against cancer, in accordance with the higher energetic demand cancer cells have to meet to keep with their accelerated expansion.^9,10^

Regarding cancer therapy, copper removal effects go beyond the energetic perspective, since disruption of copper homeostasis is also involved in changes in glycolysis,^11,12^ metastatic expansion through ATOX-ATP7A-LOX pathway^13,14^ or blood vessel formation.^15^ In addition to the classic roles of copper as a static cofactor, this transition metal can also act as a signaling or regulation agent through its dynamic binding to noncatalytic sites in proteins,^16−18^ known as labile copper. Recent studies highlight the role of labile copper in the regulation of proteins involved in cell growth and proliferation as mitogen-activated protein kinase 1 (MEK1/2) or extracellular signal-regulated kinase 1 (ERK1/2)^19,20^ Therefore, copper plays a central role in different key processes related to cancer growth, and regulating intracellular copper levels represents a promising and underexplored alternative approach for cancer treatment.

This strategy has shown promising results against triple-negative breast cancer (TNBC),^9,10,21^ reaching Phase II clinical trials using a copper chelator such as tetrathiomolybdate.^22^ TNBC cells are characterized by the lack of expression in three receptors: estrogen (ER-), progesterone (PR-), and epidermal growth factor (HER2-).^23^ This type of tumor is also characterized by its aggressiveness and poor therapeutic outcome, which is a problem of great concern considering that about 10–15% of all breast cancers are associated with TNBC.^23^ Regarding copper levels, TNBC cells also exhibit an upregulation for mitochondrial copper chaperone and cochaperone proteins (COX17 and SCO2)^24,25^ thereby suggesting an increase in copper trafficking to the mitochondria relative to healthy cells. Although copper chelation has been proven to be a promising approach to treating TNBC, it remains an underexplored horizon, especially from a nanomedicine perspective.^26^ Unlike molecular-based chelation, nanomedicine offers multiple possibilities to improve the tumor accumulation of the therapeutic vector nanoparticles over molecules, ranging from the enhanced permeability and retention effect (EPR) to an active targeting functionalization nanoparticle surface with ligands, antibodies, or cell membranes.^27,28^ To the best of our knowledge, only Cui et al.^9^ have followed this strategy using nanoparticles loaded with a copper chelator. In view of the limited number of FDA-approved medicines for TNBC,^29^ we sought to identify nanostructures with the capability of sequestration of copper applied for selective TNBC therapy.

**Figure 1:**
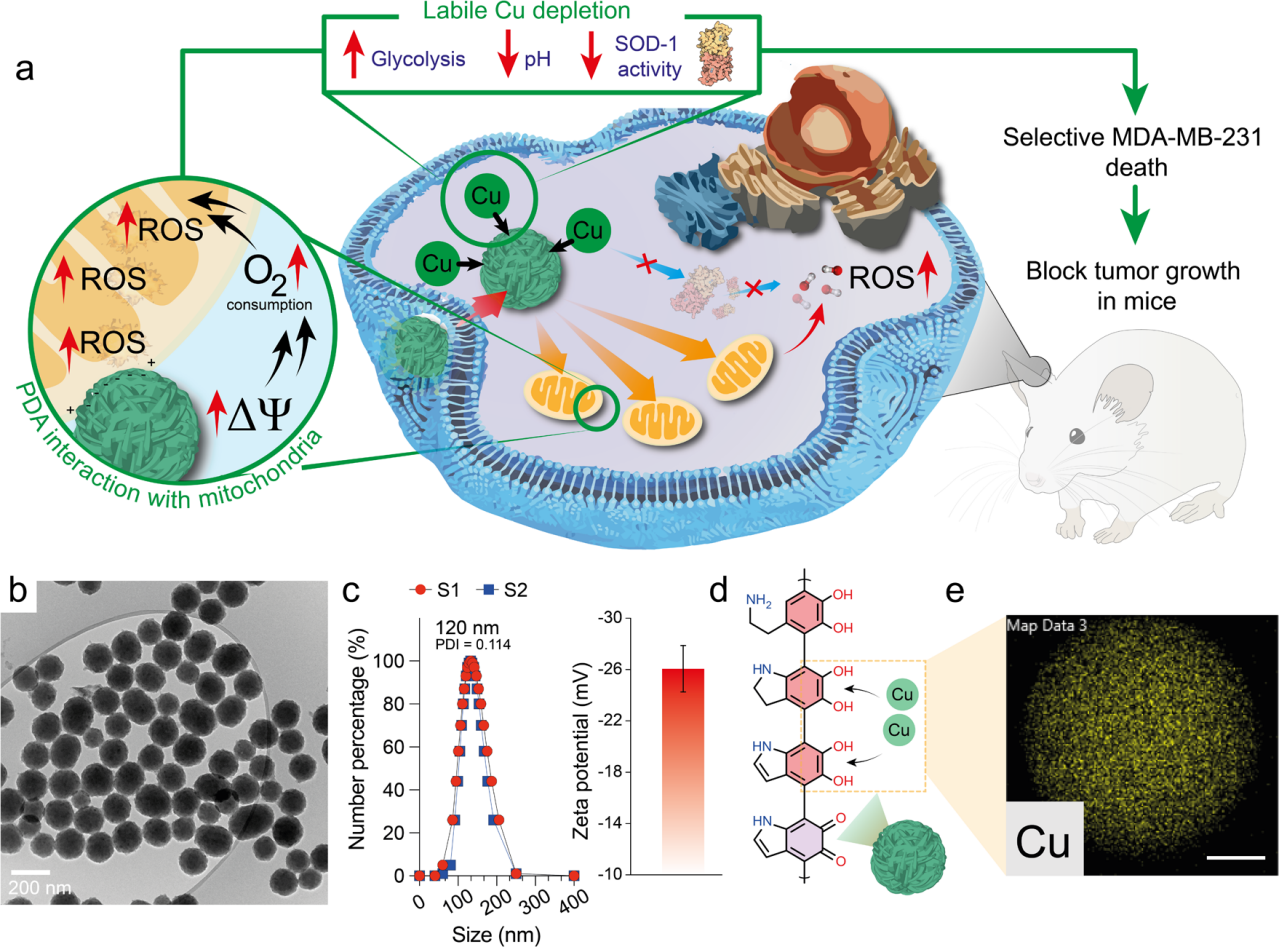
PDA nanoparticles for metal depletion as a novel anticancer strategy. (a) Polydopamine (PDA) nanoparticles can disrupt the labile copper equilibrium in MDA-MB-231 cells. Depletion of labile copper pools induces strong effects not only in metabolism but also in the activity of the key detoxifying enzyme superoxide dismutase-1 (SOD-1). After internalization, negatively charged PDA also triggered an increase in the mitochondrial membrane potential (ΔΨ), thereby affecting their metabolic activity and reactive oxygen species (ROS) levels. The combination of all these features is responsible of selective death of MDA-MB-231 breast cancer cells over MCF-10A healthy cell counterparts and is able to block tumor growth in mice. (b) TEM image of PDA nanoparticles. (c) Hydrodynamic size of two different synthesis of PDA nanoparticles. Inset: Z-potential value of PDA nanoparticles in a phosphate buffer solution (pH = 7.4, 0.1 M). (d) Chemical structure of PDA nanoparticles contains amino and catechol groups with affinity to Cu cations and is able to sequester them from solution (conditions: [PDA] = 0.1 mg mL^–1^, [Cu] = 0.1 mg mL^–1^, pH = 7.4 buffered with Tris 0.1 M). (e) EDS mapping analysis of copper in a single PDA particle after exposure to a solution containing Cu reveals its homogeneous absorption all over the PDA surface; Scale bar corresponds to 25 nm.

Given its application as a metal-removal agent,^30,31^ we envisioned polydopamine (PDA) nanoparticles as potential candidates to remove copper from TNBC cancer cells. In addition, PDA coatings have been proven to display a low affinity for abundant metals in the cell such as Na^+^, K^+^, Ca^2+^, or Mg^2+^^31^ which represents an additional advantage to increase selectivity toward cellular copper.^30,32^ So far, cancer-related research using PDA focuses on (i) its reactive oxidative species (ROS) scavenging and anti-inflammatory capabilities;^33−36^ (ii) its use in combination with plasmonic nanostructures (such as Au^37,38^ or CuS^39^) to maximize the photothermal response of the hybrid platform or (iii) its role as a vector able to load chemotherapeutics such as doxorubicin^39,40^ or paclitaxel^41^ either to perform chemotherapy. Typically, the use of PDA as a therapeutic agent relies on remote stimuli such as light or on its efficiency as a drug carrier. In contrast with these approaches, this work explores a novel strategy in cancer therapy for PDA, which is used as an efficient agent for the sequestration of intracellular labile copper, and studies its effects, ultimately leading to TNBC cell death (Figure 1a). We have demonstrated that the viability of MDA-MB-231 cells is strongly compromised by the presence of low concentrations of PDA nanoparticles, because of because of their ability to sequester copper. The use of the activity-based probe, FCP-1,^42^ enabled us to identify and quantify the depletion of labile copper pools in the presence when PDA NPs were present inside cancer cells, without altering total copper levels. In contrast, this phenomenon was less relevant for healthy breast tissue cells (i.e., MCF-10A). Furthermore, we also evaluated the influence of the PDA NPs beyond the regulation of cell metabolism. The presence of PDA NPs affected the activity of copper-dependent enzyme superoxide dismutase-1 (SOD-1). Additionally, PDA also affected mitochondria, increasing their membrane potential (ΔΨ). Both effects induced by PDA treatment in MDA-MB-231 synergistically yielded an enhanced generation of reactive oxygen species (ROS), which converged in blocking the tumor growth in xenograft-bearing mice after administration of low doses of PDA. Taken together, the findings of this study suggest that the vulnerability of TNBC to copper depletion can be leveraged for the development of more efficient, selective, and safer therapies.

## 2 Results and Discussion

### 2.1 Synthesis of PDA Nanoparticles

The synthesis of the PDA nanoparticles was conducted via polymerization of dopamine hydrochloride in a water/isopropyl alcohol mixture in basic media^43^ (see further details in the Experimental Section). TEM and dynamic light scattering (DLS) analysis revealed the formation of well-dispersed particles with a narrow size distribution around 120 nm and a PDI index of 0.114 (Figure 1b,c). Although the precise chemical structure is still under debate,^44,45^ PDA possesses different subunits within its chemical structure, mainly composed of catechol groups which exhibit a high affinity by transition metal ions including copper^30,32^ (Figure 1d). This feature was corroborated by adding 0.1 of PDA to a 0.1 mg mL^–1^ Cu solution and mapping the presence of copper in a PDA nanoparticle using STEM-EDS (Figure 1e). We systematically evaluated the affinity of PDA with other biologically relevant metals such as Fe^2+^, Ni^2+^, Mn^2+^, Co^2+^, and Zn^2+^ (Figure S1). Among all the tested metals, we found a higher affinity of PDA for Cu^2+^ and Fe^2+^ ions and a higher maximum sequestration capacity for Cu^2+^ (0.085 μg of copper per μg of PDA) compared to Fe^2+^ (0.028 μg of iron per μg of PDA). PDA nanoparticles also showed a low affinity toward other abundant ions in cells, such as Na^+^ or K^+^ (Figure S2). In terms of selectivity, PDA nanoparticles could sequester 60% of the initial amount of copper in the presence of equal concentrations of Fe^2+^, Ni^2+^, Mn^2+^, Co^2+^, and Zn^2+^ (Figure S3), and almost 80% of the initial amount of copper added to DMEM (Figure S3). All these results point out that PDA could effectively deplete intracellular labile copper.

### 2.2 PDA Nanoparticles Promote MDA-MB-231 Cell Death through Labile Copper Depletion

**Figure 2:**
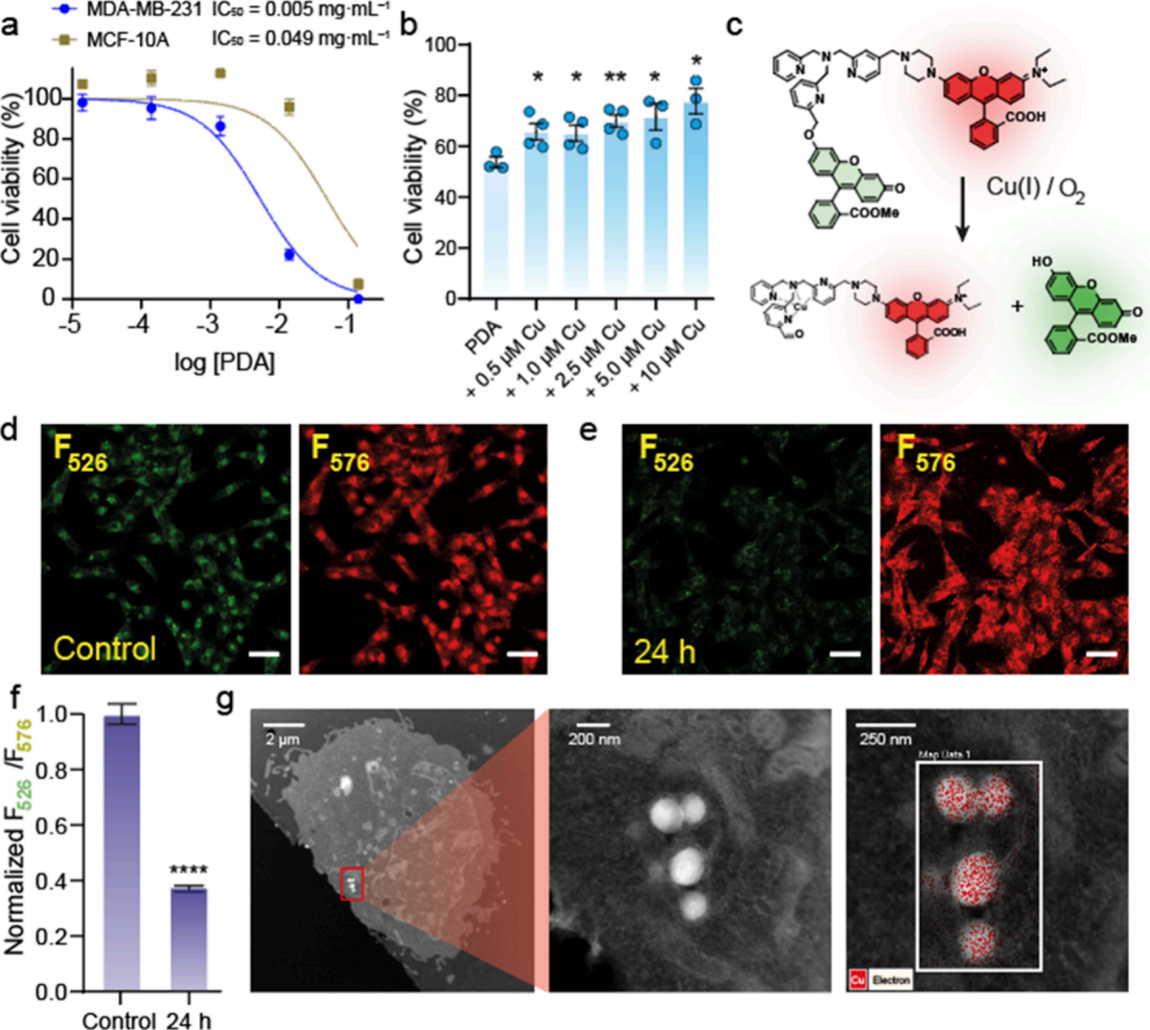
Cytotoxicity of PDA nanoparticles toward MDA-MB-231 cells is correlated with the sequestration of labile copper. (a) Comparison of the cell viability of MDA-MB-231 and MCF-10A cell lines in the presence of different concentrations of PDA NPs. (b) Cell viability of MDA-MB-231 treated with 5 × 10^–3^ mg mL^–1^ of PDA NPs with/without an external copper supplementation. (c) Mechanism of detection and quantification of labile Cu(I) using FCP-1 probe. Under an oxidative environment caused by the presence of labile Cu(I) and O_2_, cleavage of the C–O bond, which binds both fluorescent subunits, is favored. This activity-based sensing of Cu(I) cancels the FRET phenomena and is related with changes in the fluorescence spectra of the solution, which enables the detection and quantification of labile copper. (d, e) Confocal microscopy images of (d) control (i.e., no PDA nanoparticles added) and (e) treated MDA-MB-231 cells treated with 1.14 × 10^–3^ mg mL^–1^ of PDA NPs for 24 h. Fluorescence emitted at 526 nm (F_526_) by the fluorescein donor unit is acquired using the green channel, while fluorescence emitted at 576 nm (F_576_) by the rhodamine acceptor group is captured using the orange-red channel. Scale bar = 50 μm. (f) Quantification of the F_526_/F_576_ ratio fluorescence in MDA-MB-231 cells treated with 1.14 × 10^–3^ mg mL^–1^ of PDA NPs for 24 h. (g) HAADF-STEM images and EDS mapping analysis of MDA-MB-231 cells revealed the internalization of PDA NPs and the strongly increased copper levels where the NPs were present; PDA nanoparticles were incubated for 24 h with a concentration of 1.14 × 10^–3^ mg mL^–1^. The fluorescence intensity of FCP-1 was determined from experiments with an λ_ex_ of 458 nm. Scale bar = 50 μm. *P < 0.05, **P < 0.01, ***P < 0.001, and ****P < 0.0001; ns, not statistically significant. Error bars denote SEM (*n* = 8).

After evaluating the capacity of PDA NPs to sequester copper ions, we decided to evaluate their toxicity for different breast cell lines, including TNBC cell lines (MDA-MB-231 and MDA-MB-468), the non-TNBC cancer line MCF-7, and the healthy breast tissue cell line MCF-10A (Figure S4). The addition of low levels of PDA NPs dramatically decreased the cell viability in MDA-MB-231 cells; in contrast, MCF-10A cells remained viable under equivalent doses of PDA (Figure 2a). We attributed this effect to the role of PDA as a labile copper-sequestration agent, with a direct effect on the cell viability of MDA-MB-231 cells. Indeed, supplementation of cell media with CuCl_2_ augmented cell viability in the presence of PDA (Figure 2b), but no effect was detected after the addition of other biologically relevant metals such as Fe or Zn (Figure S5), indicating that copper sequestration using PDA nanoparticles selectively affects the viability of the MDA-MB-231 breast cancer cell models.

In this context, the labile copper pool is defined as the copper pool that is not tightly bound to proteins and is subjected to fast intracellular-extracellular transport.^16^ We thus sought to use activity-based sensing probes, which can detect biological analytes via selective reaction chemistry,^46,47^ focusing on metal-selective molecules designed to selectively react with the labile copper available and yield a fluorescent product.^48−50^ FCP-1 is a particularly attractive activity-based sensing probe for labile copper owing to its self-calibration properties^42^ to quantify the labile copper pool in MDA-MB-231 and MCF-10 cells after treatment with PDA. FCP-1 contains three main active units: a green-emitting (F_526_) fluorescein donor connected to a red-emitting rhodamine (F_576_) acceptor through a Tris(2-pyridylmethyl)amine (TPA), which acts as a binding site for labile Cu(I) (Figure 2c). When they are part of the same molecule, rhodamine quenches fluorescein fluorescence through fluorescence resonance energy transfer (FRET) phenomena and gives rise to orange-red fluorescence, but in the presence of the Cu(I)/O_2_, the C–O bond between fluorescein and TPA is oxidatively cleaved,^42^ disabling the FRET phenomena and giving rise to more green fluorescence. Thus, the sequestration of labile Cu can be directly related to a decay in green fluorescence from the fluorescein moiety (F_526_) and thereby a decrease in the F_526_/F_576_ ratio^42^ (Figure 2d–f).

After 24 h of incubation with PDA nanoparticles, we could detect a drop of 65% in the F_526_/F_576_ ratio in MDA-MB-231 cells (Figure 2d–f, bright field images can be found in Figure S6). In contrast, just 20% was observed for MCF-10A cells (Figure S7, bright field images can be found in Figure S8). The total copper levels for both MDA-MB-231 and MCF-10A remained unvaried, thereby indicating that copper was sequestered intracellularly but not released by the NPs (Figure S9). HAADF-STEM images combined with EDS mapping analysis further confirmed the successful internalization of PDA NPs within the cytosol of MDA-MB-231 cells (Figure S10). It also confirmed the high and selective colocalization of copper where the PDA NPs were located (Figures 2g and S11). In addition, the copper signal was barely discernible outside the surface of the PDA nanoparticle (Figure S12). These multiple lines of data suggest that sequestration of intracellular labile copper by PDA NPs is taking place, with a clear correlation to a selective decrease in the MDA-MB-231 cancer cell viability over healthy MCF-10A counterparts.

### 2.3 PDA Treatment Promotes Disruption of Redox Homeostasis and Metabolism in MDA-MB-231 Cells

**Figure 3:**
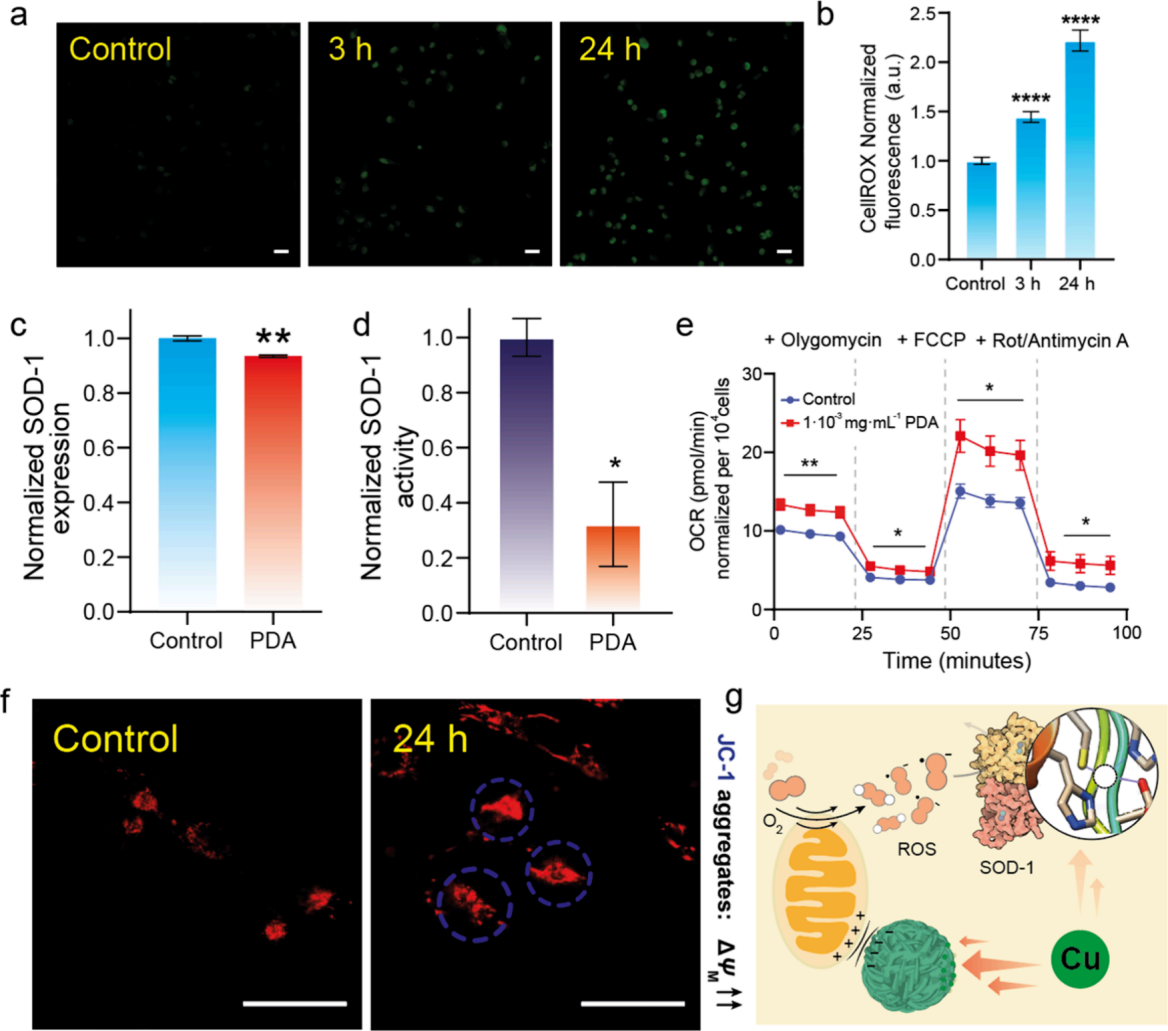
Disruption of redox homeostasis and metabolism driven by PDA in MDA-MB-231 cells. (a, b) Confocal microscopy analysis of reactive oxygen species (ROS) in MDA-MB-231 cells treated with 5 × 10^–3^ mg mL^–1^ using fluorescent probe CellRox at different incubation times. Fluorescence intensity of the CellRox probe was determined from experiments with λ_ex_ = 485 nm. Scale bar = 50 μm. Results are normalized to control fluorescence = 1. Error bars denote SEM (*n* = 8). (c) Quantification of superoxide dismutase-1 (SOD-1) expression by Western blot in MDA-MB-231 cells treated with 5 × 10^–3^ mg·mL^–1^ of PDA for 24 h, using glyceraldehyde 3-phosphate dehydrogenase as the control protein. Error bars denote SEM (*n* = 3). (d) Determination of SOD-1 protein activity in MDA-MB-231 cells after treatment with 5 × 10^–3^ mg mL^–1^ PDA for 24 h. Error bars denote SEM (*n* = 3). (e) Oxygen consumption rate (OCR) analysis of MDA-MB-231 cells treated with different concentrations of PDA for 24 h. The measurement was performed by a Seahorse analyzer, by adding 1 μM of oligomycin after 28 min, carbonyl cyanide p-(trifluoromethoxy) phenylhydrazone (FCCP) (1 μM) after 54 min and a 1:1 mixture of rotenone A/antimycin A (0.5 μM) after 80 min. Error bars denotes SEM (*n* = 9). (f) Confocal microscopy analysis of mitochondrial membrane potential (ΔΨ) using JC-1 fluorescent probe after the treatment of MDA-MB-231 cells incubated with 1.14 × 10^–3^ mg mL^–1^ of PDA for 24 h. The increase in red fluorescence by JC-1 indicates a major state of aggregation within the mitochondrial membrane due to its higher membrane potential. JC-1 aggregates were excited with 535 nm with an Ar laser. Scale bar = 50 μm. (g) Schematic summary of the influence of PDA in redox homeostasis and metabolism in MDA-MB-231 cells. PDA promotes the depletion of labile copper, one of the metal cofactors of SOD-1, thus blocking its activity toward ROS detoxification. On the other hand, PDA treatment unbalances mitochondrial ΔΨ which can be correlated to an increase in OCR and subsequent ROS production. *P < 0.05, **P < 0.01, ***P < 0.001, and ****P < 0.0001; ns, not statistically significant. Error bars denote SEM (*n* = 8).

Given the potent cytotoxicity of PDA nanoparticles toward MDA-MB-231 cells, we investigated the mechanisms behind the observed cell death phenotype. First, we explored the influence of PDA treatment in redox homeostasis by analyzing intracellular reactive oxygen species (ROS) generation using CellROX as a common fluorescent probe. Indeed, we could detect a time-dependent increase in ROS levels after the treatment of MDA-MB-231 cells with 5 × 10^–3^ mgmL^–1^ PDA NPs (Figure 3a,b). In addition, we observed a downregulation of α-tubulin expression after PDA treatment of MDA-MB-231 cells (Figure S13), which has previously been attributed to ROS generation.^51,52^ PDA-induced generation of ROS was unexpected since several reports support the notion that PDA nanoparticles have antioxidant activity in different cell lines, including human gingival epithelial cells^35^ or fibroblasts.^36^ Indeed, the treatment of MCF-10A cells with an analogous dose of PDA resulted in an ROS scavenging effect (Figure S14). Ex vitro ROS generation experiments demonstrated the key role of metal chelation by PDA nanoparticles in the activation of O_2_ and H_2_O_2_ (Figure S15). Therefore, we posit that the metal availability, which may be different for different cell lines, may determine the amount of metal sequestered by PDA nanoparticles and the in situ production of different ROS.

At this point, we focused on establishing a correlation between the decrease in the labile copper pools induced by PDA and ROS generation. This idea might be counterintuitive since a rise in labile copper levels is typically related to oxidative stress;^8^ however, we considered another possibility that may involve mechanisms that prevent cells from removing already existing ROS. Along these lines, copper is an essential cofactor of the enzyme SOD-1, one of the most common antioxidant enzymes present in the cytosol of eukaryotic cells.^53^ Although the changes in expression of SOD-1 were minimal after PDA treatment of MDA-MB-231 cells (Figures 3c and S16), its intrinsic activity was decreased by a factor of almost three (Figure 3d). Similar results were obtained by Cui et al.^9^ after treating MDA-MB-231 cells using a copper-selective chelator. We hypothesize that the copper scarcity scenario induced by the internalization of PDA NPs in MDA-MB-231 cells blocks the delivery of copper to SOD-1 enzyme, presumably through the CCS chaperone for the superoxide dismutase enzyme,^53^ and as a consequence, the produced ROS cannot be removed.

We then decided to study mitochondrial respiration, as one of the major sources of ROS in cells,^54^ to investigate possible changes triggered by the presence of PDA nanoparticles. Measurement of the oxygen consumption rate (OCR) by a Seahorse analyzer indicated a significant rise in the basal OCR after the treatment of MDA-MB-231 cells with PDA nanoparticles (Figure 3e). Moreover, the addition of the mitochondrial membrane uncoupling drug carbonyl cyanide-4 (trifluoromethoxy) phenylhydrazone (FCCP), during the experiment revealed large differences in the maximum respiration between treated and control MDA-MB-231 cells (Figure 3e). In contrast, no significant effects could be detected for the corresponding treatment in MCF-10A cells (Figure S17). These results suggest that the treatment with PDA NPs may affect the mitochondrial membrane potential (Δψ) of MDA-MB-231 cells. The hyperpolarization of the mitochondria can also be related to the increase in ROS production,^55^ and would be in agreement with the results displayed in Figure 3a,b. By using the fluorescent dye JC-1, we analyzed the Δψ of mitochondria from treated MDA-MB-231 cells (Figures 3f and S18). The JC-1 dye mechanism of action is based on its aggregation-forming dimers with red emission within mitochondria with a high Δψ, whereas a decrease in Δψ favors its monomeric state with green emission.^56^ Together with the OCR results, the addition of PDA resulted in a higher red fluorescence of JC-1, while the green fluorescence slightly decreased in the case of PDA-treated MDA-MB-231 cells, thereby indicating that PDA was indeed inducing a higher Δψ in MDA-MB-231 cells (Figure 3f).

Additionally, we investigated the effects of PDA treatment on the metabolism of MDA-MB-231 and MCF-10A cells, respectively. By analyzing the extracellular acidification rate (ECAR) it is possible to determine the source of the produced ATP and determine potential metabolism changes induced by PDA NPs. In the case of MDA-MB-231 cells, the basal levels of ECAR were significantly higher than in the control experiment (Figure S19) in contrast to the absence of effect for MCF-10A cells (Figure S20). These results were indicative of a change toward a more glycolytic metabolism under the presence of PDA nanoparticles in MDA-MB-231 cells. A similar trend was reported by Ishida et al.^11^ in different cancer cells including BxPC3, SUIT2, MDA-MB-157, and SKOV3. They quantified both an important increase in lactate levels and upregulation of AMP-activated protein kinase (AMPK) under copper chelation conditions, indicating an enhanced glycolytic pathway^11^ which indeed was analogous to our observations with PDA NPs. To quantify the ATP production rate and its specific source, we measured both the ECAR and the OCR in PDA-treated cells under the addition of specific mitochondrial unit inhibitors. Changes in pH can be directly related to the lactate and ATP (glycoATP), generated in the glycolysis pathway. In the case of mitochondrial-ATP (mitoATP), it can be determined through OCR measurement as O_2_ is required for the biosynthesis of ATP in mitochondria. By adding oligomycin to inhibit ATP synthase and rotenone/antimycin A to inhibit complex I and IV of mitochondria, respectively, it was possible to finally differentiate the sources of produced ATP (Figure S21). We could quantify a 1.56-fold increase in production of ATP via glycolysis and a 1.47-fold increase in ATP production via phosphorylation oxidation in agreement with the increased basal OCR in treated cells (Figure S22, further details of calculations can be found in the Experimental Section).

Taking into account all of the data, we posit that PDA nanoparticles can induce oxidative stress in MDA-MB-231 cells through the simultaneous generation of ROS via increasing Δψ and through depletion of labile copper and the subsequent loss of activity of the antioxidant enzyme, SOD-1 (Figure 3g). Additionally, depletion of copper affects ATP production in MDA-MB-231, which changes toward glycolysis after PDA treatment. The combination of the impossibility of detoxifying mitochondrial ROS and its enhanced generation led to cell apoptosis, as shown by an increase in cell viability after the addition of 10 μM apoptosis rescuer Z-VAD-FMK (Figure S23).

### 2.4 Therapeutic Efficacy of PDA and Metal Analysis *In Vivo*

**Figure 4:**
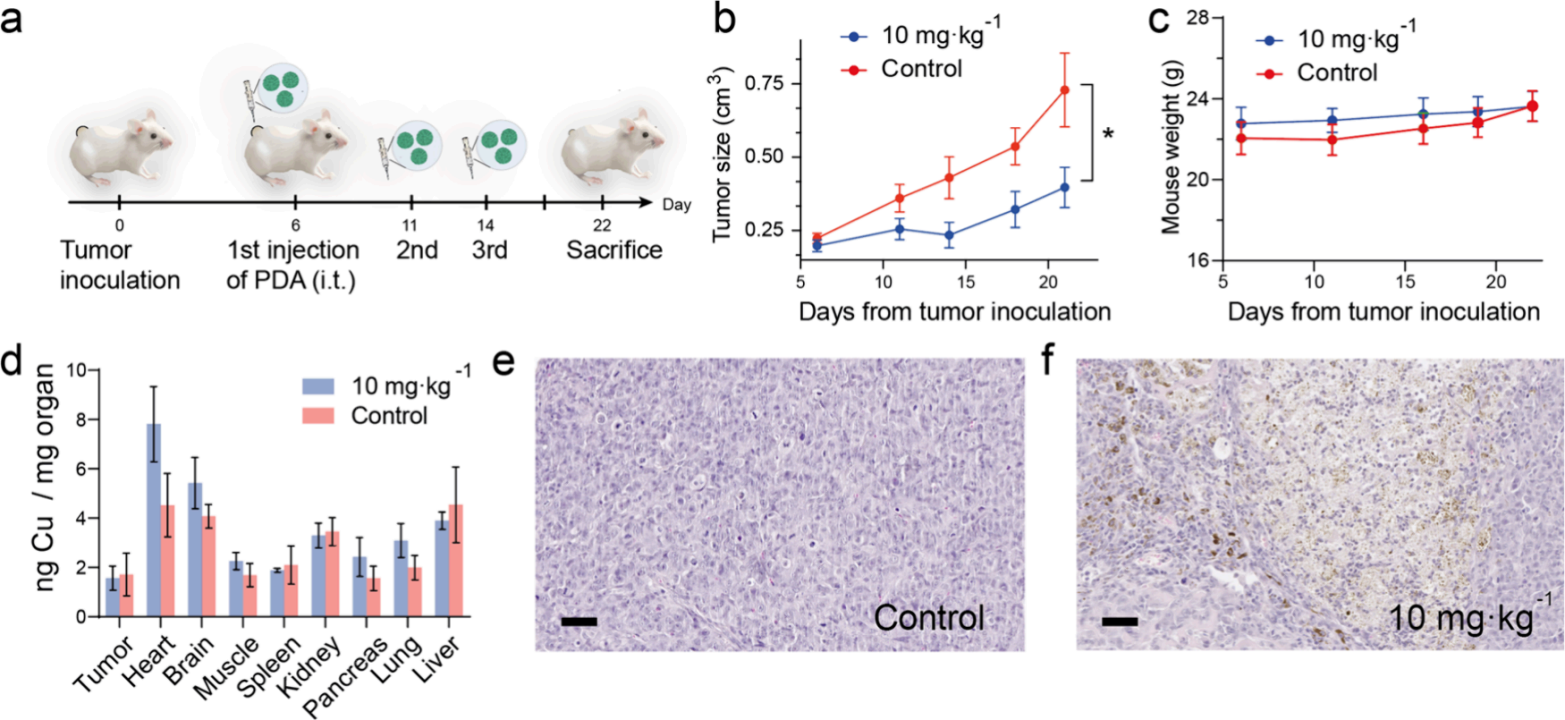
*In vivo* therapeutic efficacy of PDA nanoparticles in a MDA-MB-231 mouse tumor model. (a) Treatment strategy for therapeutic efficacy of PDA NPs study in MDA-MB-231 tumor-bearing mice. The treated group comprised 9 mice that received 3 intratumoral doses of 10 mg kg^–1^ PDA NPs on days 6, 11, and 14 after tumor inoculation. (b) Relative tumor volume (*V*/*V*_0_) evolution with time in control and treated groups. Error bars denote SEM (*n* = 8 for control group and *n* = 9 for treated group). (c) Monitoring of mouse weight throughout the experiment in the control and treated groups. Error bars denote SEM (*n* = 8 for control group and *n* = 9 for treated group). (d) Analysis of copper levels in different organs (expressed as nanograms of metal/mg organ) in control (red bar) and treated groups (blue bar). All differences between control and 10 mg kg^–1^ group were non significant (omitted in the plot for clarity). H&E staining of tumor tissue in (e) control and (f) treated mice. Scale bar = 20 μm. Error bars denote SEM (*n* = 3). No statistically significant differences were found *P < 0.05, **P < 0.01, ***P < 0.001, and ****P < 0.0001; ns, not statistically significant.

Encouraged by the remarkable cytotoxicity of PDA nanoparticles toward MDA-MB-231 cells and their low toxicity toward MCF-10A cells, we investigated the antitumor effect of the PDA nanoparticles *in vivo* using a MDA-MB-231 breast cancer model in nude mice. In the treatment group, mice with MDA-MB-231 tumors received intratumoral injections of PDA nanoparticles at a dose of 10 mg kg^–1^ on days 6, 11, and 14 after tumor inoculation (*n* = 9), while the control group received a saline solution (Figure 4a). The PDA treatment was initiated when the tumor volume reached 180 mm^3^. It can be seen that PDA treatment significantly hindered tumor progression and growth 22 days after tumor inoculation. Tumors in the control group exhibited a considerable increase in size and weight, reaching a relative tumor size of 3.0, whereas the treated group demonstrated a restrained growth with tumor volumes reaching 2.0, underscoring the therapeutic efficacy of PDA nanoparticles (Figures 4b and S24). The treatment did not produce significant weight loss among the mice (Figure 4c).

Furthermore, we conducted an analysis of the total content of biologically relevant metals (Zn, Fe, and Cu) in various organs, including the tumor, heart, brain, muscle, spleen, kidney, pancreas, lung, and liver. However, our analysis revealed no significant changes in the total copper levels in any of the examined organs. This result aligns well with the ICP-MS data obtained *in vitro* (Figure S6) and further supports our description of the role of PDA as able to sequester labile copper, without depleting total copper levels (Figure 4d). Analogous results were obtained after the analysis of other biologically relevant metals such as iron (Figure S25) and zinc (Figure S26), indicating that PDA treatment did not alter the total concentration of metals inside the treated cells. Hematoxylin and eosin (H&E)-stained images of tumor tissue (Figure 4e,f) revealed important damage to tumor tissue in mice administered with an intratumoral injection of 10 mg kg^–1^ of PDA, while no apparent damage could be observed in other major organs as kidney, spleen (Figure S27), heart, pancreas (Figure S28), lungs, or liver (Figure S29), supporting the good biosafety properties of the PDA treatment.

## 3 Conclusions

PDA nanoparticles represent an attractive nanomedicine platform able to selectively affect the viability of triple-negative breast cancer cells compared to their healthy breast tissue cell counterparts through the modulation of labile copper levels and the induction of oxidative stress. We could track the specific depletion of labile copper levels using the activity-based probe FCP-1, induced by the treatment with PDA NPs in MDA-MB-231 cells without affecting total copper pools. The metal-chelating groups present on the surface of PDA NPs could effectively sequester labile copper present in MDA-MB-231 cells, a process that we have been able to link to their selective cell death.

Mechanistic investigations revealed that PDA NPs increased reactive oxygen species (ROS) levels through two synergistic mechanisms. First, labile copper sequestration led to the inactivation of superoxide dismutase 1, an enzyme that relies on copper for its antioxidant activity. Second, PDA NPs increase the mitochondrial membrane potential, which likely contributes to enhanced ROS production. Taken together, these findings highlight the ability of PDA NPs to disrupt redox homeostasis and trigger oxidative-stress-mediated apoptosis in triple-negative breast cancer cells. Furthermore, we validated the therapeutic efficacy of the PDA NPs *in vivo* by showing that they can block tumor growth in a mouse model. Notably, the administration of PDA NPs as a single-agent nanomedicine led to a significant reduction in tumor volume by a factor of 1.66 after 22 days of administration, without additional treatments or the use of external light or heat activation. Overall, this study establishes that nanomedicines that deplete the labile copper pool can be leveraged to selectively target cancer cells over normal cells by exploiting this metal as a key nutrient disease vulnerability. Moreover, our findings provide valuable insights into the mechanisms of action of PDA NPs through inducing oxidative stress while hampering the cell response mechanisms, features that can be exploited in the context of designing new therapeutics that regulate copper-dependent cell growth and proliferation (i.e., cuproplasia^8,16^) and copper-dependent cell death (i.e., cuproptosis^16,57^).

## 4 Experimental Section

### 4.1 Instruments

Confocal fluorescence imaging was performed with a Zeiss laser scanning microscope LSM880 with a 20x dry objective lens using Zen 2015 software (Carl Zeiss, Zen 2.3 black). Metal content in cells was determined by measuring ^63^Cu and ^64^Zn using a Thermo Fisher iCAP-Qc ICP-MS instrument in KED mode. Aristar BDH Ultra Concentrated nitric acid to dissolve samples was purchased from VWR. *In vitro* OCR and ECAR measurements were performed using a Seahorse XF96 analyzer. The superoxide dismutase activity test was purchased from Fisher Scientific (Reference number 15665069).

### 4.2 Chemicals and Materials

Dopamine hydrochloride (DA·HCl, Sigma-Aldrich), CuCl_2_ (>99.0%, Sigma-Aldrich), FeSO_4_ (98%, Alpha Aesar), ZnCl_2_ (≥98.0%, Sigma-Aldrich), FeCl_2_·2H_2_O (98%), MnCl_2_ (>98%), ZnCl_2_ (>99%), NiCl_2_ (>99%), CoCl_2_ (>99%), NaCl (>98%), KCl (>98%), 1,3-diphenylisobenzofuran (DPBF, 97%), 3,3′,5,5′-tetramethylbenzidine (>98%), 9,10-anthracenediyl-bis(methylene)dimalonic acid (ABDA, >90%), 2-isopropanol, (2-PrOH, > 90%, VWR), NH_4_OH (J.T. Baker, 14.8 M in water), phosphate buffer saline (PBS without Ca and Mg, Corning), Dulbecco’s modified Eagle’s medium (DMEM, (GlutaMax)), Hanks’ balanced salt solution (HBSS + CaCl_2_, + MgCl_2_, Gibco), Cell Counting Kit-8 (CCK-8, Dojindo), CellROX-Green reagent (InvitroGen), JC-1 (Thermofisher Scientific), Agilent Seahorse XF Calibrant (pH = 7.4), Seahorse XF Base medium without phenol red, Seahorse XF 1.0 M glucose solution, Seahorse XF 100 mM pyruvate solution, Seahorse XF 200 mM glutamine solution, Cell MitoStress, and ATP-rate cell kits were purchased from Agilent Technologies.

### 4.3 Polydopamine Nanoparticle (PDA) Synthesis

A protocol adapted from Nieto et al. was used.^43^ Briefly, 42.5 mL of 2-PrOH, 90 mL of milli-Q water, and 3.8 mL of NH_4_OH were added to a round-bottom flask, and the solution was stirred for 30 min. Then, 500 mg of DA·HCl were dissolved in 10 mL of milli-Q water and added dropwise. The mixture was stirred overnight at room temperature. The product was isolated by centrifugation (10,000 rpm, 10 min, two cycles). To quantify the concentration of PDA nanoparticles, 100 μL of nanoparticle solution in distilled water was added to a vial and was left to dry overnight (oven, 60 °C). The obtained mass difference was divided by a volume of 100 μL to determine the PDA concentration. For *in vitro* and *in vivo* experiments, the final product was resuspended in PBS and maintained at room temperature for further use.

### 4.4 Metal Sequestration Experiments

0.05 mg mL^–1^ of PDA nanoparticles were incubated with a variable amount of metal ion (added as metal chloride) for 2 h at 37 °C in distilled water. Then, samples were filtered using a 0.22 μm Nylon filter to remove the PDA nanoparticles with the metals absorbed. The metal concentration in the resulting supernatant was determined using MP–AES. The metal mixture was prepared by adding 20 μL of each 1 mM MCl_2_ solution and then adding 0.05 mg mL^–1^ PDA nanoparticles. DMEM experiment was prepared by adding 20 μL of 1 mM CuCl_2_ to 1 mL of DMEM and then adding 0.05 mg mL^–1^ PDA nanoparticles.

### 4.5 Cell Viability Experiments

All cells used in this research were maintained by the UC Berkeley Tissue Culture Facility. MDA-MB-231, MDA-MB-468, and MCF-7 were seeded in 75% of confluency in 96-well cell plates prior to a day before the experiments. In the case of MCF-10A, 1 × 10^5^ cells were seeded. PDA nanoparticles from stock solution in PBS were added to DMEM to achieve a final concentration of PDA of 1.14 × 10^–5^, 1.14 × 10^–4^, 1.14 × 10^–3^, 1.14 × 10^–2^ or 1.14 × 10^–1^ mg mL^–1^. Before the addition, the PDA nanoparticle stock solution was sonicated for 20 min. Then, a small fraction from the stock solution was irradiated with UV light before each experiment to avoid bacteria contamination. After 24 h, wells were washed with PBS (1×) and 100 μL of 10% v/v CCK-8 in DMEM solution were added to the well. Cell plates were incubated at 37 °C in a 5% CO_2_ incubator for 3 h (in the case of MDA-MB-231, MDA-MB-468, and MCF-7 cells) or 3.5 h (in the case of MCF-10A cells). Finally, the absorbance at 450 nm was measured by using a plate reader. Viability is presented as the percentage of control (*n* = 3 ± SEM). For recovery experiments using Cu, Fe, and Zn, cells were seeded under the same conditions as in the cell viability experiment. Stock solutions of CuCl_2_, FeSO_4,_ and ZnCl_2_ were prepared by dissolving the metal salts in H_2_O to a final concentration of 0.70 mM, and then subsequently in DMEM to a final concentration of 35 μM. PDA was added to a final concentration of 5.0 × 10^–3^ mg mL^–1^ and incubated for 24 h. Cell viability was measured following the same protocol mentioned before. Absorbance at 450 nm was measured using a BioTek Synergy MX Microplate Reader. For the apoptosis experiment, apoptosis inhibitor Z-VAD-FMK was coincubated with PDA particles in 10 μM and 1.14 × 10^–3^ mg mL^–1^ for 24 h. Cell viability was measured using the same protocol as that mentioned before.

### 4.6 Intracellular Labile Copper Analysis Using FCP-1

FCP-1 was synthesized following previous reports.^2^ MDA-MB-231 and MCF-10A cells were seeded in 60% confluency in a Chamber slide 8-well. PDA nanoparticles resuspended in DMEM were added to a final concentration of 1.14 × 10^–3^ mg mL^–1^ and incubated for 24 h. Wells were washed with HBSS once, and then, FCP-1 was added to a final concentration of 1 μM (0.6% of DMSO). Before imaging, cells were incubated for 30 min at 37 °C in a 5% CO_2_ incubator. FCP-1 was excited at 458 nm with an Ar laser, and the emissions were collected using a META detector between 465 and 541 nm (F_525_), and between 559 and 710 nm (F_575_). Results are expressed considering average F_525_/F_575_ of control = 1 ± SEM (*n* = 8).

### 4.7 ICP-MS Analysis of MDA-MB-231 and MCF-10A Cells

MDA-MB-231 or MCF-10A cells were seeded in six-well plates in 75% confluency. PDA nanoparticles resuspended in DMEM were added to a final concentration of 1.14 × 10^–3^ mg mL^–1^ and incubated for 3 and 24 h. Then, the wells were washed with ice-cold PBS (1×) three times. Finally, 350 μL of concentrated nitric acid was added to the plate and the mixture was incubated for 48 h at room temperature. Before analysis, 20 ppb of Ga was added to each sample as an internal standard. Results are expressed considering the average Cu/Zn of control = 1 ± SEM (*n* = 6).

### 4.8 OCR and ECAR Measurements

First, 5 × 10^4^ cells from the MDA-MB-231 or MCF-10A cell line were seeded in Agilent Seahorse XF96 Cell Culture microplates. PDA nanoparticles were added to a final concentration of 1.14 × 10^–3^ mg mL^–1^ and incubated with cells for 24 h at 37 °C in a 5% CO_2_ incubator. For the mitochondrial stress assay, cells were washed two times using freshly prepared Seahorse XF DMEM medium (10 mM glucose, 1 mM pyruvate, and 2 mM glutamine) and incubated with this medium for 1 h at 37 °C in a non-CO_2_ incubator. OCR and ECAR were measured under basal conditions and after the sequential addition of oligomycin (1 μM, added after 28 min), FCCP (1 μM, added after 54 min), and Rotenone/Antimycin A (0.5 μM, added after 80 min) (*n* = 9 per condition for OCR and ECAR for each cell line). For the ATP-rate assay, cells were washed once with Seahorse XF DMEM medium (same composition as mitochondrial-stress assay) and incubated for 1 h at 37 °C in a non-CO_2_ incubator. Before measurement, cells were washed once using Seahorse XF DMEM medium. OCR and ECAR were measured under basal conditions and after the sequential addition of oligomycin (1 μM, added after 28 min) and Rotenone/Antimycin A (0.5 μM, added after 54 min) (*n* = 9 per condition for OCR and ECAR for each cell line). The total cell number per well was determined using methylene blue (MB). Briefly, after the Seahorse measurement was done, the cell medium was removed, replaced with PBS with 0.5% MB solution, and incubated overnight at room temperature. The plate was protected from light using film. The next day, the MB solution was removed, and the plate was washed with distilled water until the supernatant was completely clear of MB. The plate was then left at room temperature to dry out. Finally, 100 μL of Coomassie destain solution (40% MeOH, 4% acetic acid) was added to each well. The plate was left at room temperature with gentle mixing for 10 min. Finally, 80 μL from each well was transferred to a fresh 96-well plate and the absorbance at 668 nm was measured using the plate reader. The cell number was then normalized to the control experiment.

The mitoATP (i.e., the mitochondrial ATP production) value was calculated as follows:
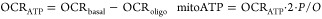
where OCR_ATP_ is the oxygen consumption rate associated with ATP production, OCR_basal_ is the OCR before the addition of oligomycin or rotenone/antimycin A and OCR_oligo_ is the OCR after the addition of oligomycin. OCR_basal_ and OCR_oligo_ are values obtained experimentally derived from Figure S17. P/O is the ratio of oxygen atoms employed in ATP production and has a value of 2.45.^1^

The glycoATP (i.e., the glycolytic ATP production) with value was calculated as follows:
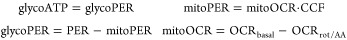
where glycoPER is the proton efflux rate associated with glycolytic ATP production, PER is the total proton efflux rate (experimentally obtained from the extracellular acidification rate measurement), mitoPER is the mitochondrial proton efflux rate, mitoOCR is the oxygen consumption rate associated with the mitochondria, and OCR_rot/AA_ is the oxygen consumption rate after addition of rotenone/antimycin A mixture. CCF is the CO_2_ contribution factor and has a value of 0.60.

### 4.9 Reactive Oxygen Species (ROS) Analysis Using CellROX

MDA-MB-231 and MCF-10A cells were seeded in 60% confluency in a Chamber slide 8-well. PDA nanoparticles resuspended in DMEM were added to a final concentration of 5 × 10^–3^ mg mL^–1^ and incubated for 24 h. Wells were washed with HBSS once, and then CellRox reagent was added to a final concentration of 5 μM (1% DMSO). Cells were incubated for 30 min at 37 °C in a 5% CO_2_ incubator. Then, wells were washed using HBSS twice, and fixed with paraformaldehyde (3.7%) for 15 min at 37 °C prior to imaging. CellROX was excited with 485 nm with an Ar laser, and the emissions were collected using a META detector between 500 and 540 nm. Results are normalized to control fluorescence = 1 ± SEM (*n* = 8).

### 4.10 ROS Analysis Using DPBF, ABDA, and TMB

For the DPBF experiment, 0.05 of PDA or 0.05 mg mL^–1^ loaded with copper and iron was added to a 0.12 mM DPBF solution in EtOH:H_2_O (2:1). For ABDA, 0.05 of PDA or 0.05 mg mL^–1^ loaded with copper and iron was added to a 0.12 mM ABDA solution in H_2_O. Control experiments using solutions only containing the probe were conducted to avoid self–degradation of the probe. For TMB, 10 μL of a stock TMB solution of 5 mg mL^–1^ in DMSO were mixed with 0.05 of PDA or 0.05 mg mL^–1^ loaded with copper and iron in H_2_O and varying concentrations of H_2_O_2_ were added. pH of the reaction was controlled using a phosphate buffer (pH = 6.5). Solutions were incubated at room temperature in darkness, and UV–vis spectra of the solution were recorded at different reaction times.

### 4.11 Reactive Oxygen Species (ROS) Analysis Using CellROX

MDA-MB-231 and MCF-10A cells were seeded in 60% confluency in a Chamber slide 8-well. PDA nanoparticles resuspended in DMEM were added to a final concentration of 5 × 10^–3^ mg mL^–1^ and incubated for 24 h. Wells were washed with HBSS once, and then CellRox reagent was added to a final concentration of 5 μM (1% DMSO). Cells were incubated for 30 min at 37 °C in a 5% CO_2_ incubator. Then, wells were washed using HBSS twice, and fixed with paraformaldehyde (3.7%) for 15 min at 37 °C prior to imaging. CellROX was excited with 485 nm with an Ar laser, and the emissions were collected using a META detector between 500 and 540 nm. Results are normalized to control fluorescence = 1 ± SEM (*n* = 8).

### 4.12 *In Vitro* Mitochondrial Membrane Potential Using JC-1

MDA-MB-231 and MCF-10A cells were seeded in 60% confluency in a Chamber slide 8-well. PDA nanoparticles resuspended in DMEM were added to a final concentration of 1.14 × 10^–3^ mg mL^–1^ and incubated for 24 h. Wells were washed with HBSS once, and then, the JC-1 reagent was added to a final concentration of 10 μg mL^–1^ (1% DMSO). Cells were incubated for 10 min at 37 °C in a 5% CO_2_ incubator. Then, the wells were washed using HBSS once prior to imaging. JC-1 monomers and aggregates were excited with 450 and 535 nm with an Ar laser, respectively, and the emissions were collected using a META detector between 570 and 620 nm.

### 4.13 Western Blot Analysis

MDA-MB-231 Cells were treated with 5.0 × 10^–3^ mg mL^–1^ PDA nanoparticles for 24 h. Cells were washed using cold PBS (1×) twice. Finally, cells were collected using a cell scratch and centrifuged at 6000 rpm, 20 min at 4 °C. The supernatant was discarded, and cell pellets were stored at −80 °C. A 150 μL portion of RIPA buffer (Thermo, cat. no. 89900) was added to cell pellets and incubated for 30 min. The protein concentrations were quantified by a Pierce BCA assay (Thermo Scientific, 23250). The GAPDH antibody was purchased from Cell Signaling Technologies (Asp175, no. 9661) and used with a dilution of 1:3000. The SOD-1 antibody was purchased from Santa Cruz Biotechnologies (sc-101523) and used with a dilution of 1:250. α-tubulin antibody was purchased from Cell Signaling Technology (3873S) and used with a dilution of 1:1000. Blots were first treated with GAPDH and SOD-1 antibodies to reveal these proteins. Then, the blots were stripped (20 min, room temperature) and treated with α-tubulin antibody. Results are normalized by GAPDH expression (*n* = 3).

### 4.14 SOD-1 Activity Analysis

MDA-MB-231 cells were seeded in six-well plates with 75% confluency. PDA nanoparticles dissolved in DMEM were added to the well in a concentration of 5 × 10^–3^ mg mL^–1^ and incubated for 24 h. Then, cells were trypsinized and centrifuged at 250 g for 10 min at 4 °C and washed with ice-cold PBS under the same conditions. Cells were sonicated and finally centrifuged at 1500 g for 10 min at 4 °C. The supernatant was diluted 1:4 and analyzed using the SOD-1 activity test. Ten microliters of the diluted samples were added to a 96-well plate, followed by the addition of 50 μL of substrate solution and 25 μL of Xanthine Oxidase solution. The mixture was incubated for 20 min at room temperature. Finally, the absorbance at 450 nm was measured using a plate reader.

### 4.15 *In Vivo* Therapeutic Efficacy of PDA

The procedures performed in this study were previously approved under Project License PI44/21 by the Ethic Committee for Animal Experiments from the University of Zaragoza (Comisión Ética Asesora para la Experimentación Animal de la Universidad de Zaragoza). Mice were fed ad libitum, and their maintenance and care under specific pathogen-free conditions were performed according to the Spanish Policy for Animal Protection RD53/2013 and the European Union Directive 2010/63 regarding the protection of animals destined for experimental and other scientific purposes. In this study, six-to eight-week-old female nude-Foxn1nu (Envigo) were used. All the animals were maintained for 7 days under quarantine as soon as they arrived at the animal facilities and before starting the experiments. For the induction of the xenograft tumor, animals received a subcutaneous injection of 5 × 10^6^ MDA-MB-231 cells in 200 μL of PBS. To evaluate the potential weight loss or distress symptoms, animals were weighed and monitored every 2 days. Tumor sizes were measured with a caliper every 2 days. The manipulation of the animals was always performed under sterile conditions in a hood. After 5 days of tumor implantation, a dose of 10 mg·kg^–1^ PDA NPs were administered via intratumoral injection, once the tumor volume reached 180 mm^3^. Euthanasia of animals was performed by CO_2_ inhalation. Selected organs were collected from each animal for histopathological analysis. For histopathological analysis the samples were fixed in 4% paraformaldehyde (Alfa Aesar) for 24 h, followed by cold 70% ethanol. Tissue samples were then embedded in paraffin and three-micrometer sections were stained with H&E.

### 4.16 Metal Analysis of Organs by MP-AES

The collected organs were carefully processed to assess the copper, iron, or zinc contents in each of them. First, the major organs were weighed, and then they were placed in 15 mL centrifuge tubes. In each tube, 2 mL of aqua regia was added for digestion purposes. However, considering their larger size, the livers were placed in 50 mL centrifuge tubes and treated with 5 mL of Aqua Regia. After complete digestion, the mass of copper and iron in each organ was determined using Agilent 4100 MP-AES analysis. The obtained values were then normalized by considering both the total volume of the solution and the weight of the respective organ. By employing this approach, we were able to evaluate the copper and iron levels present in the collected organs accurately and reliably.

### 4.17 Statistical Analysis

All the results are expressed as mean ± SEM. Statistical analysis of the biological experiments and the significant differences among the means were evaluated by two-way analysis of variance (ANOVA) for multiple comparisons by Dunnett’s multiple comparisons test using GraphPad Software. In the case of OCR and ECAR measurements, each row (i.e., each data collected at different times) was statistically compared using grouped *t*-tests. Statistically significant differences were expressed as follows: *p < 0.05, **p < 0.005, ***p < 0.0005 and ****p < 0.00005.

## Data Availability

Research data will be provided upon reasonable request to the corresponding authors.

## Supporting Information Available

Affinity study of PDA to different cationic species, viability tests on cancer and healthy cells, confocal analysis, intracellular copper levels, advanced electron microscopy characterization, western blot analysis, ROS tests, and *in vivo* studies (PDF)
