# Supplementary material for: Nanomedicine Targeting Cuproplasia in Cancer: Labile Copper Sequestration Using Polydopamine Particles Blocks Tumor Growth In Vivo through Altering Metabolism and Redox Homeostasis
Source: ACS Appl Mater Interfaces. 2024 Jun 3;16(23):29844–55. doi: 10.1021/acsami.4c04336 (PMC11181271; doi:10.1021/acsami.4c04336)
Supplement: Supplementary file 1 — am4c04336_si_001.pdf [file am4c04336_si_001.pdf]

Supporting Information

Nanomedicine Targeting Cuproplasia in Cancer: Labile Copper Sequestration using Polydopamine Particles Blocks Tumor Growth in vivo through Altering Metabolism and Redox Homeostasis

Javier Bonet-Aleta[1,2,3,4], Miguel Encinas-Gimenez[1,2,3,5], Miku Oi[4], Aidan T. Pezacki[4], Victor Sebastian[1,2,3,5], Alba de Martino[6], Ana Martín-Pardillos[,1,2,3,5], Pilar Martin-Duque[2,5,7], Jose L. Hueso[1,2,3,5], Christopher J. Chang[4,8,9], Jesus Santamaria[1,2,3,5]*,*

[1] Instituto de Nanociencia y Materiales de Aragon (INMA) CSIC-Universidad de Zaragoza, Campus Rio Ebro, Edificio I+D, C/ Poeta Mariano Esquillor, s/n, 50018, Zaragoza, Spain

[2] Networking Res. Center in Biomaterials, Bioengineering and Nanomedicine (CIBER-BBN), Instituto de Salud Carlos III; 28029 Madrid, Spain

[3] Department of Chemical and Environmental Engineering, University of Zaragoza, Campus Rio Ebro, C/María de Luna, 3, 50018 Zaragoza, Spain

[4] Department of Chemistry, University of California, Berkeley, CA, 94720, United States

[5] Instituto de Investigación Sanitaria (IIS) de Aragón, Avenida San Juan Bosco, 13, 50009 Zaragoza, Spain

[6] Instituto Aragonés de Ciencias de la Salud (IACS); Instituto de Investigación Sanitaria (IIS) de Aragón, Avenida San Juan Bosco, 13, 50009 Zaragoza, Spain

[7] Instituto de Salud Carlos III (ISCIII). Departamento de Desarrollo de Medicamentos y Terapias Avanzadas. Ctra. de Pozuelo, 28, 28222 Majadahonda, Madrid, Spain

[8] Department of Molecular and Cell Biology, University of California, Berkeley, CA, 94720, United States

[9] Helen Willis Neuroscience Institute, University of California, Berkeley, CA, 94720, United States

E-MAIL: chrischang@berkeley.edu , jesus.santamaria@unizar.es

SUPPLEMENTARY FIGURES

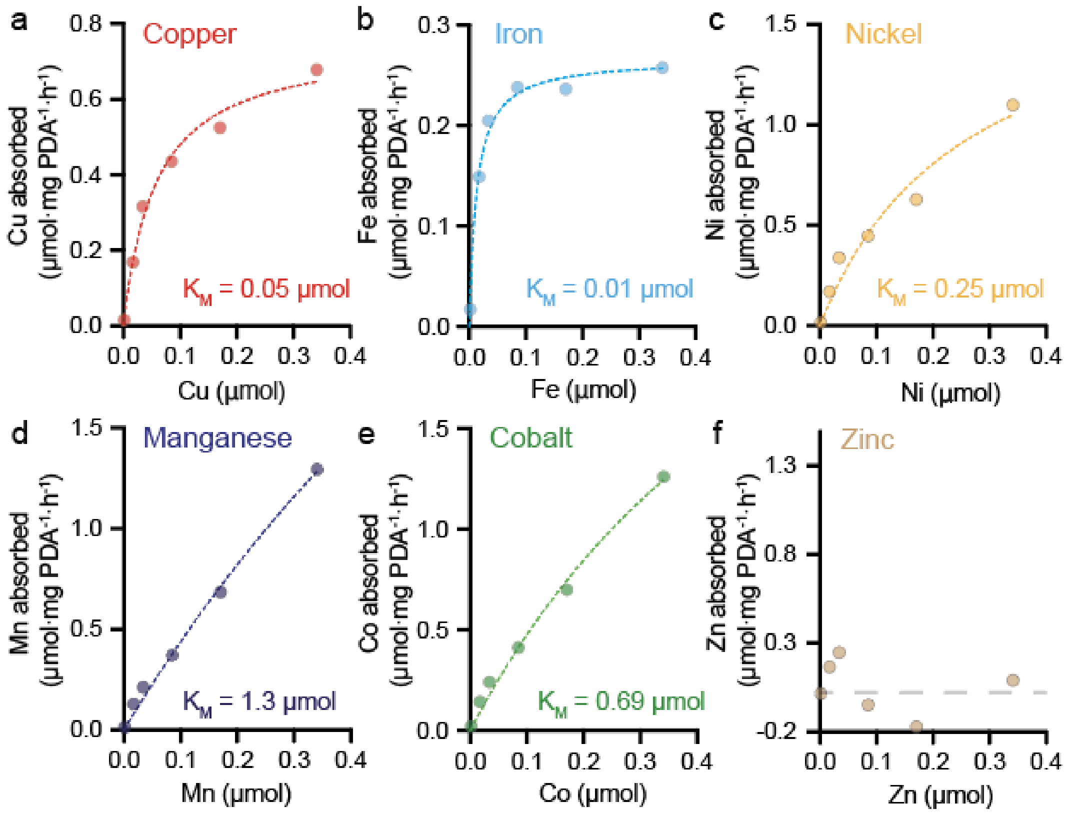

Supplementary Figure S1. Affinity of PDA nanoparticles by different divalent transition metal ions, including (a) copper, (b) iron, (c) nickel, (d) manganese, (e) cobalt and (f) zinc. Experimental conditions: [PDA] = 0.05 mg·mL^{-1}, T = 37 ºC. Incubation time: 2h. Total volume = 1 mL.

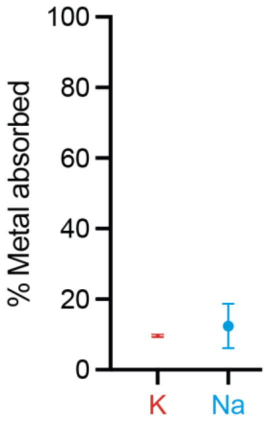

Supplementary Figure S2. Amount of K^+ or Na^+ absorbed by PDA nanoparticles. Experimental conditions: [PDA] = 0.05 mg·mL^{-1}, T = 37 ºC, [M] = 1 mM. Incubation time: 2h.

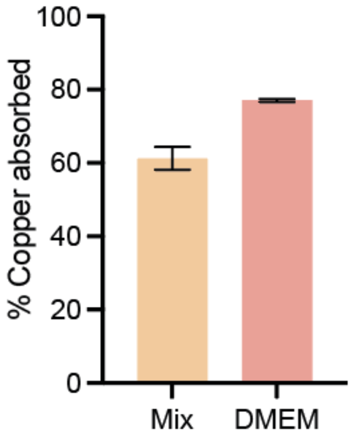

Supplementary Figure S3. Amount of copper absorbed by PDA nanoparticles under different conditions. Mix corresponds to a solution containing 20 μM of $CuCl_2$, 20 μM of $FeCl_2$, 20 μM of $MnCl_2$, 20 μM of $CoCl_2$, 20 μM $ZnCl_2$ and 20 μM $NiCl_2$. DMEM corresponds to a cell medium solution supplemented with 20 μM of CuCl2. Incubation time: 2 h, T = 37 ºC, [PDA] = 0.05 mg·mL–1.

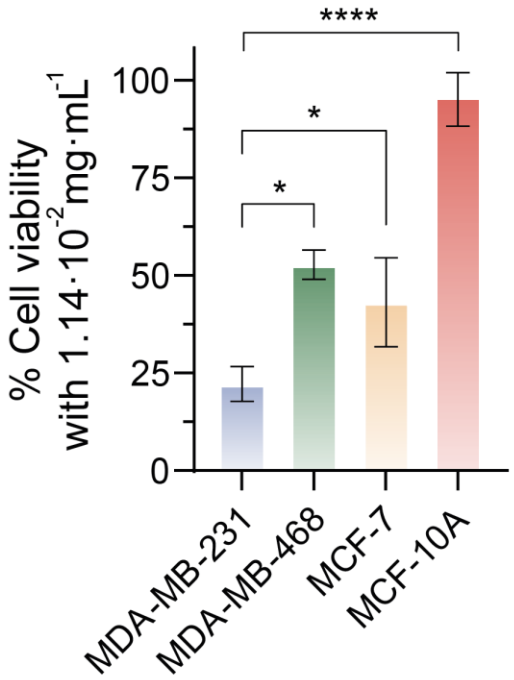

Supplementary Figure S4. Viability of different cell lines after 24 h of incubation with $1.14 \cdot 10^{-2}$ $mg \cdot mL^{-1}$ of PDA. Results revealed that breast cancer cells (MDA-MB-231, MDA-MB-468, and MCF-7) reduced viability compared to healthy cells (MCF-10A) after treatment with PDA particles. Results are expressed as Mean ± S.E.M, n =3 biological independent experiments. *P < 0.05, **P < 0.01, ***P < 0.001, and ****P < 0.0001; ns, not statistically significant.

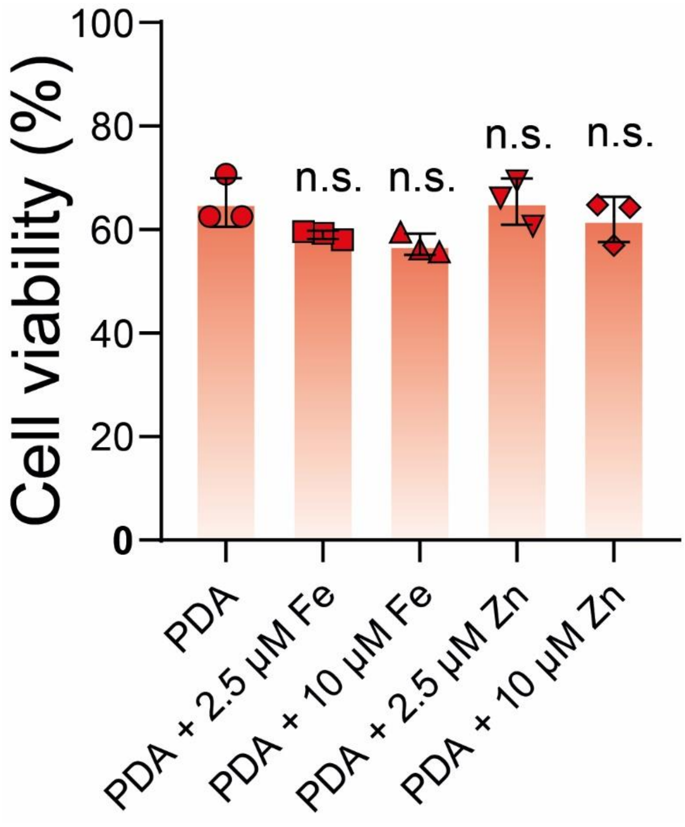

Supplementary Figure S5. Cell viability of MDA-MB-231 treated with $5 \cdot 10^{-3}$ mg·mL^{-1} of PDA NPs with an external supplementation of zinc and iron. Results are expressed as Mean ± S.E.M, n =3 biological independent experiments. *P < 0.05, **P < 0.01, ***P < 0.001, and ****P < 0.0001; ns, not statistically significant.

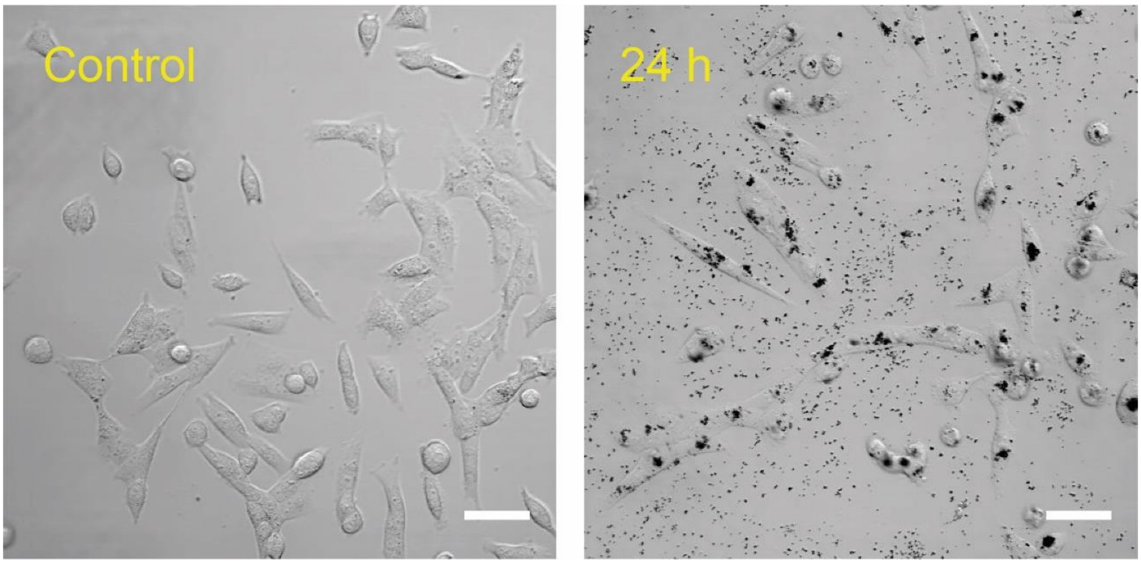

Supplementary Figure S6. Bright field images of control (left) and PDA-treated (right) MDA-MB-231 cells with $1.14 \cdot 10^{-3}$ $mg \cdot mL^{-1}$ after 24 h. Scale bar = 50 μm.

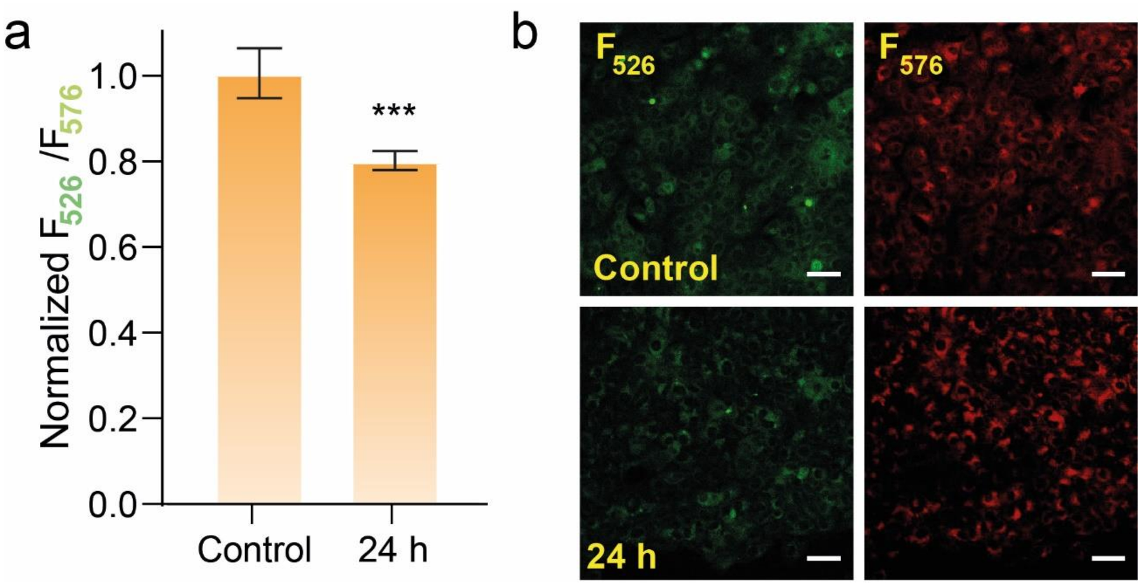

Supplementary Figure S7. (a) Quantification of F_{526}/F_{576} ratio and (b) confocal microscopy images of control and treated MCF-10A cells treated with $1.14 \cdot 10^{-3}$ mg·mL^{-1} of PDA NPs for 24 h. Scale bar = 50 μm. Error bars denotes S.E.M (n = 8). *P < 0.05, **P < 0.01, ***P < 0.001, and ****P < 0.0001; ns, not statistically significant.

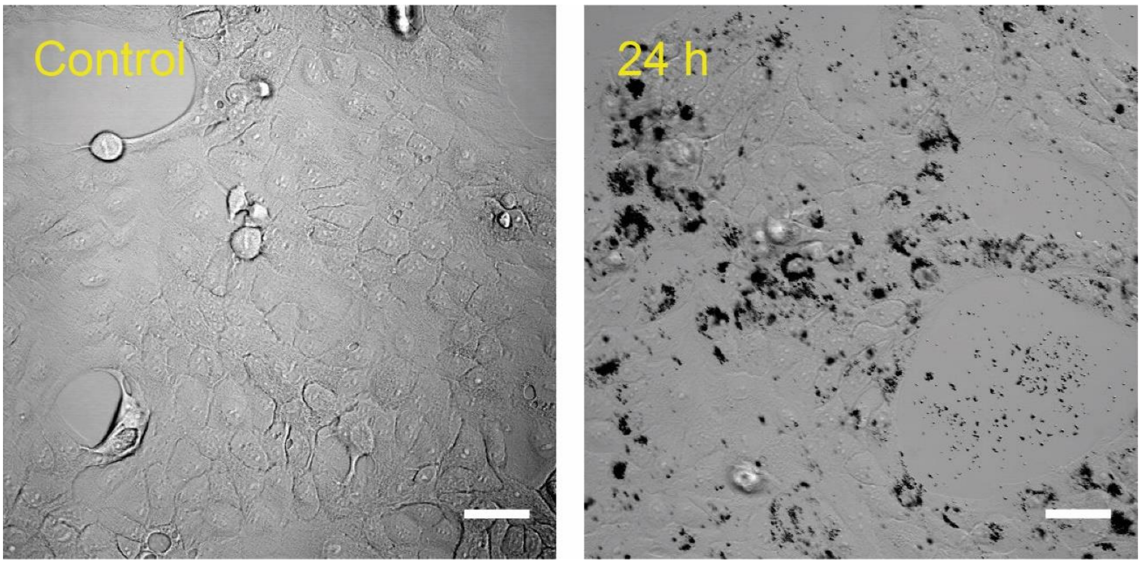

Supplementary Figure S8. Bright field images of control (left) and PDA-treated (right) MCF-10A cells with $1.14 \cdot 10^{-3}$ $mg \cdot mL^{-1}$ after 24 h. Scale bar = 50 μm.

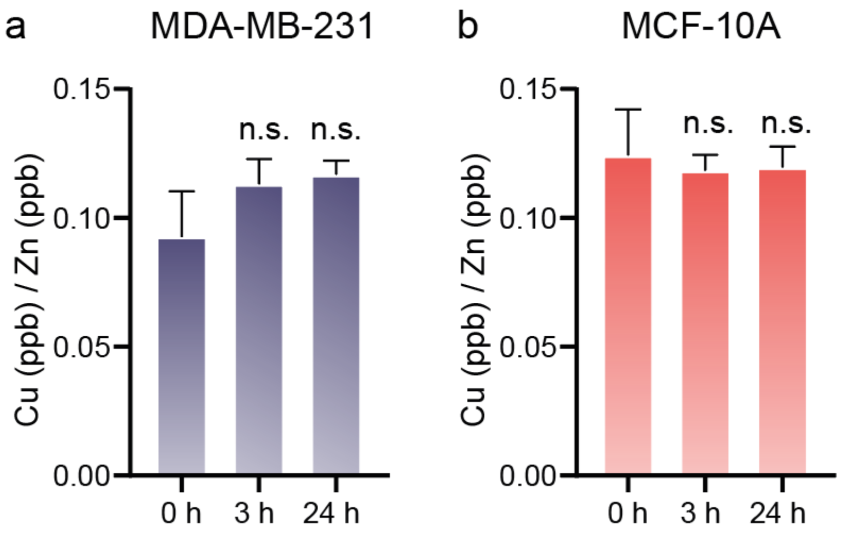

Supplementary Figure S9. ICP-MS analysis of intracellular copper levels in (a) MDA-MB-231 and (b) MCF-10A cells after the treatment with $1.14 \cdot 10^{-3}$ $mg \cdot mL^{-1}$ of PDA for 3 h and 24 h. Results are expressed as $^{63}Cu/^{64}Zn$ to normalized by number of cells based on the cellular ^{64}Zn content. Error bars denote S.E.M, n=6 biological independent experiments. ns, not statistically significant.

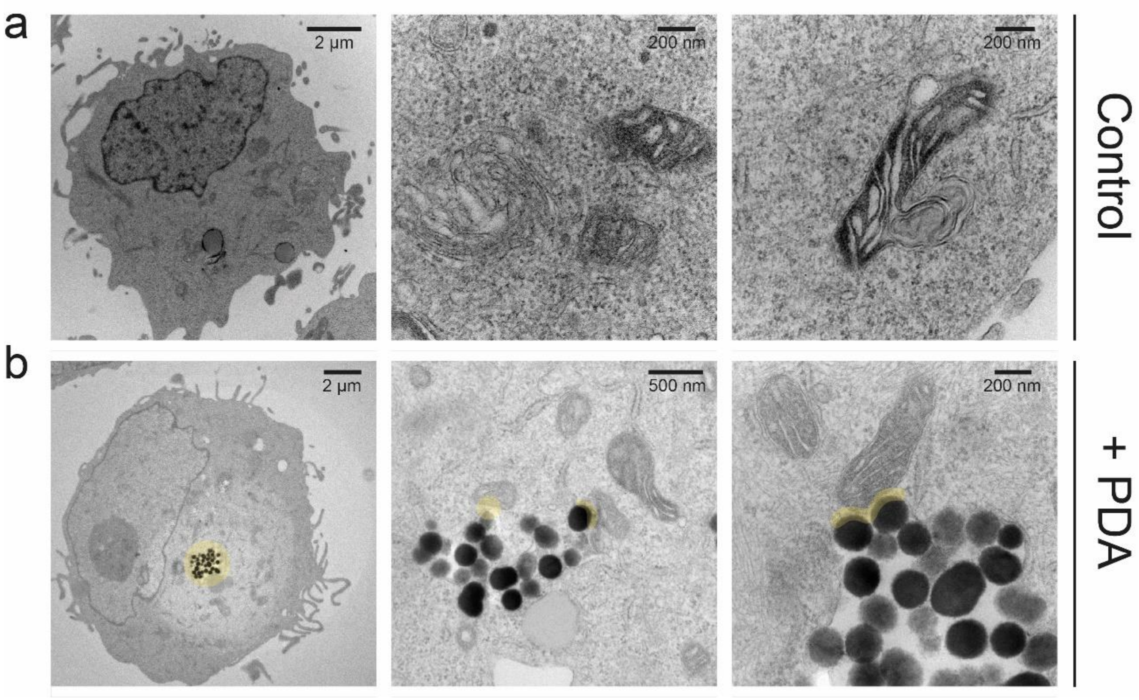

Supplementary Figure S10. Representative transmission electron microscopy (TEM) images of (a) control MDA-MB-231 cells and (b) treated MDA-MB-231 cells with $1.14 \cdot 10^{-3}$ $mg \cdot mL^{-1}$ of PDA for 24 h.

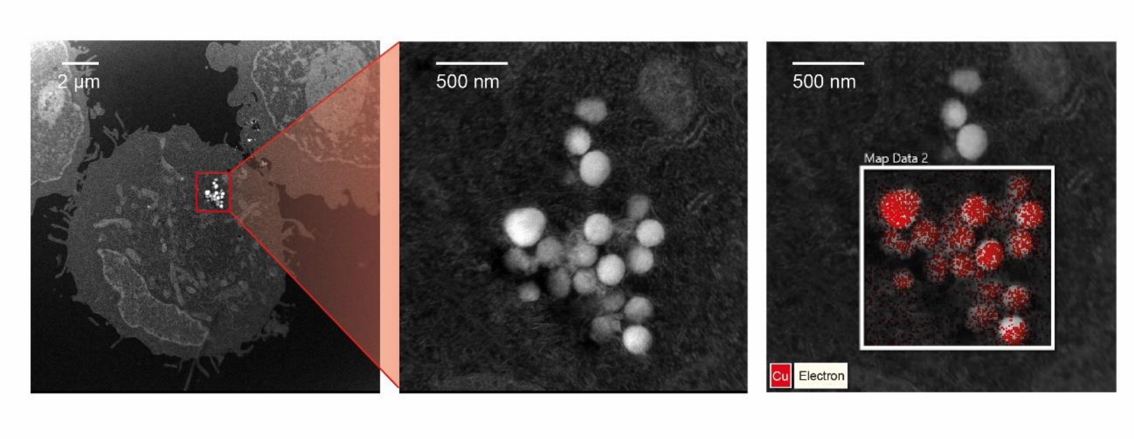

Supplementary Figure S11. STEM-EDS-HAADF images of treated MDA-MB-231 cells revealed their presence inside the cells and the accumulation of copper inside the PDA nanoparticles after 24 h of incubation with a concentration of $1.14 \cdot 10^{-3}$ mg·mL^{-1}.

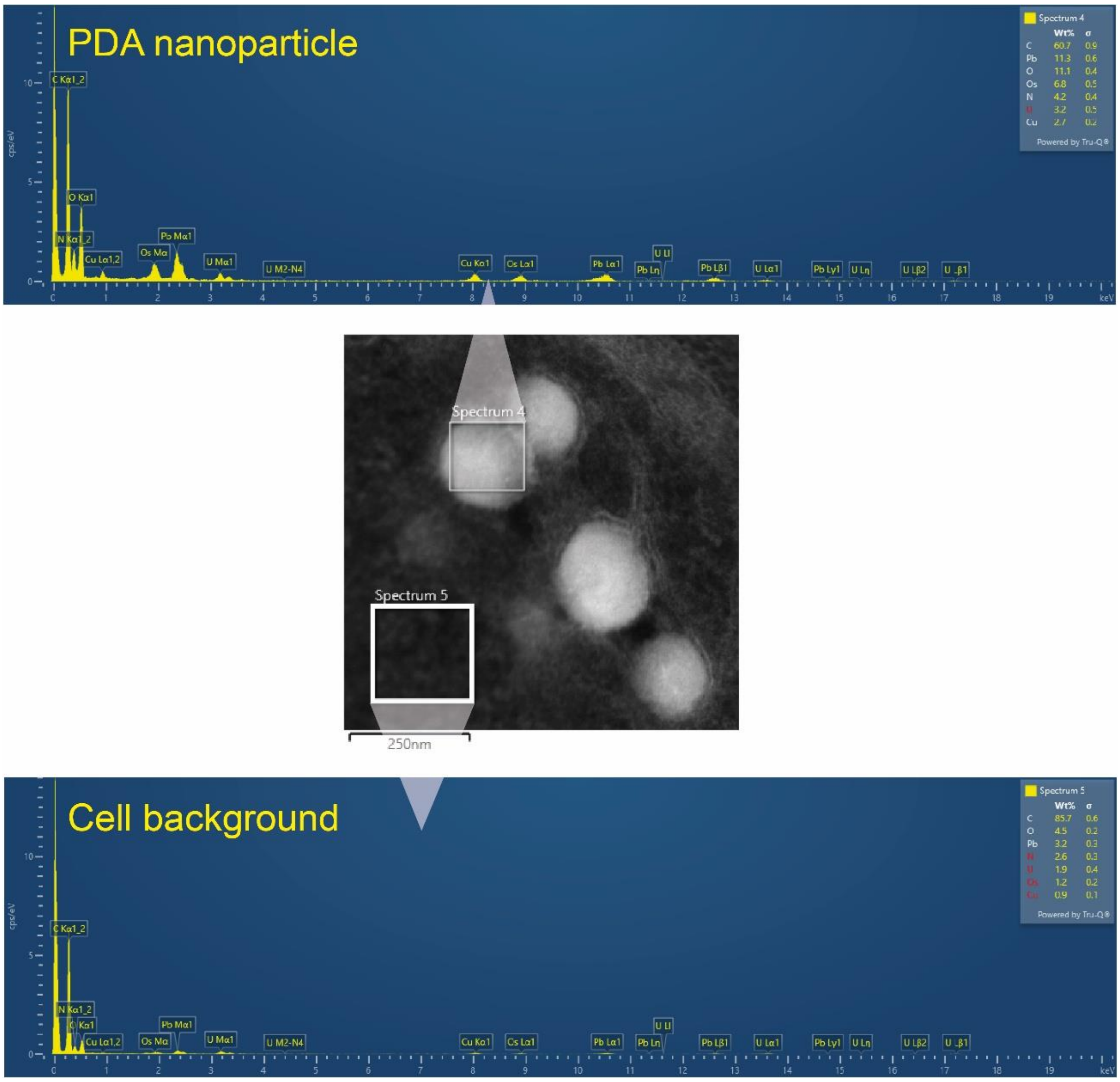

Supplementary Figure S12. EDS spectra of the surface of a single internalized PDA nanoparticle revealed a 2.7 % of copper, while the background signal presents only a residual amount of copper.

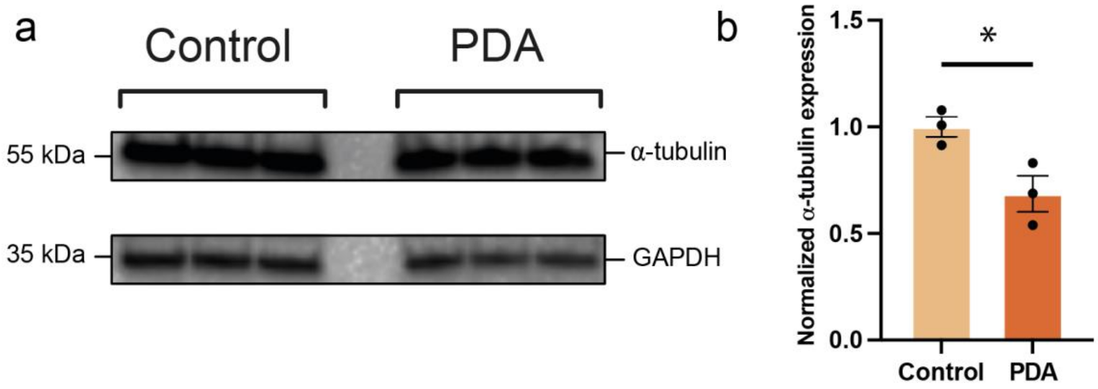

Supplementary Figure S13. (a) Western-blot analysis and (b) quantification of α-tubulin and Glyceraldehyde 3-phosphate dehydrogenase (GAPDH) proteins in MDA-MB-231 cells treated $5.0 \cdot 10^{-3}$ $mg \cdot mL^{-1}$ of PDA for 24 h. *P < 0.05, **P < 0.01, ***P < 0.001, and ****P < 0.0001; ns, not statistically significant. Error bars denote S.E.M (n = 3).

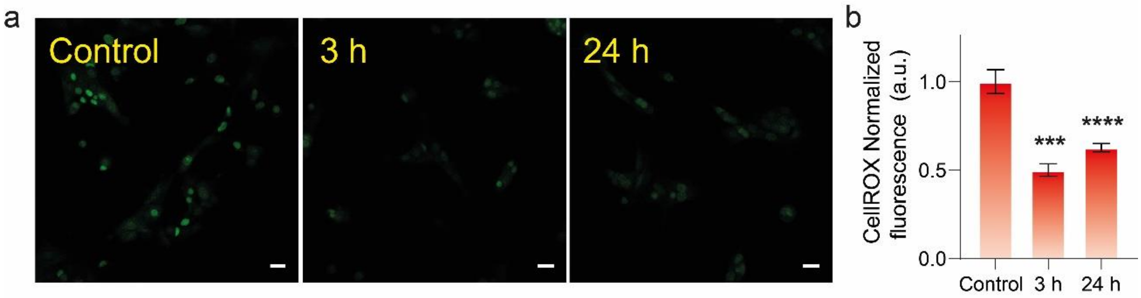

Supplementary Figure S14. (a) Confocal microscopy analysis and (b) quantification of ROS present in MCF-10A cell using CellROX™ fluorescent probe after the treatment with $5.0 \cdot 10^{-3}$ mg·mL^{-1} of PDA revealed its role quenching intracellular ROS. λ_{ex} = 485 nm. Scale bar = 50 µm. Error bars denote S.E.M, n=8 biological independent experiments. *P < 0.05, **P < 0.01, ***P < 0.001, and ****P < 0.0001; ns, not statistically significant.

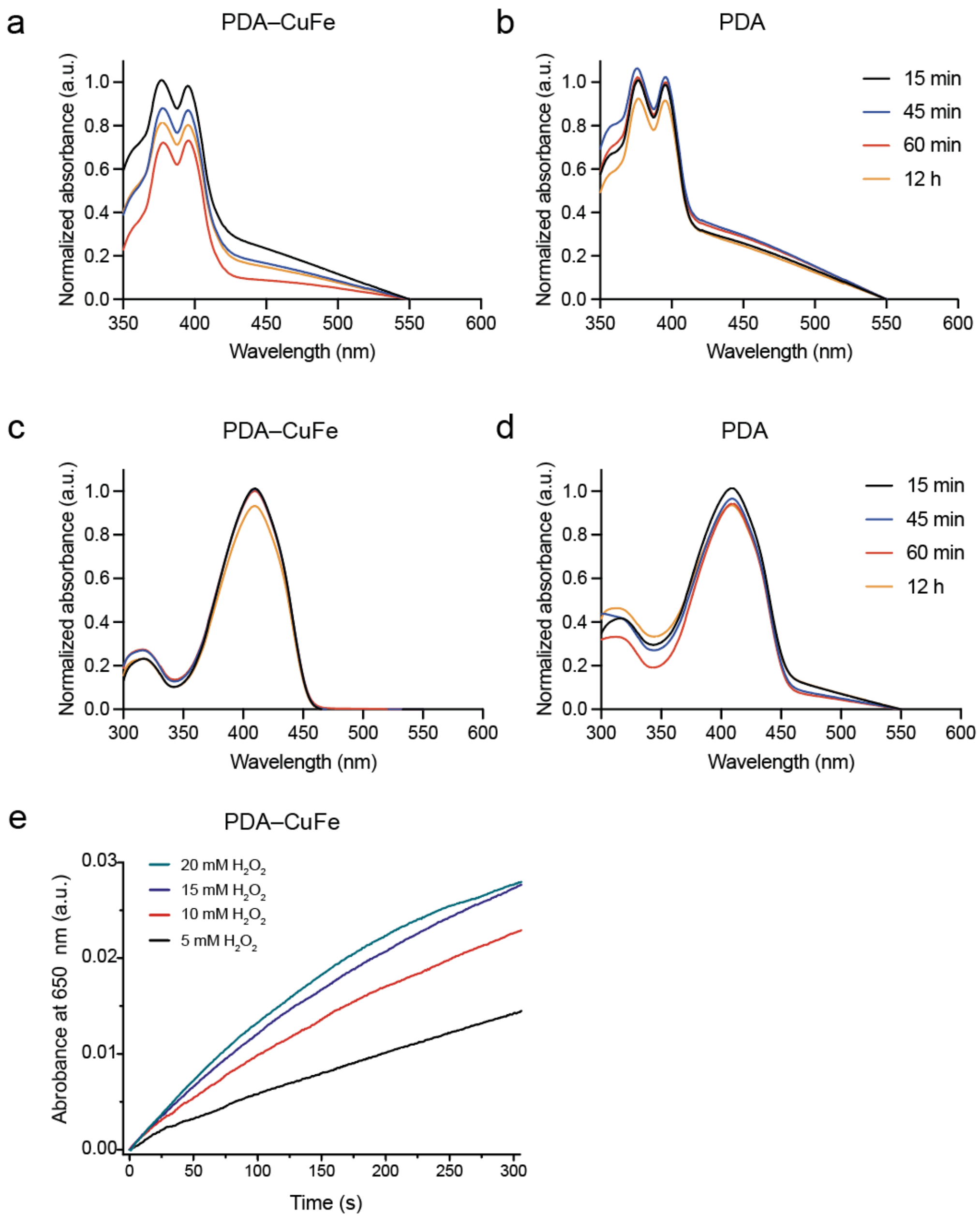

New Supplementary Figure S15. Activation of O_2 or H_2O_2 with (a, c, e) PDA nanoparticles loaded with Fe^{2+} and Cu^{2+} ions or (b, d) bare PDA nanoparticles. (a–b) UV–vis. spectra of ABDA at different reaction times (c–d) UV–vis. spectra of DPBF at different reaction times. (e) Evolution of the absorbance at 650 nm corresponding to the generation of oxidized TMB indicating an activation of H_2O_2 by PDA–CuFe. Reaction conditions: T = 25ºC, pH = 6.5 (buffered with Na_2HPO_4/NaH_2PO_4 100 mM), [PDA] = 0.05 mg·mL^{-1}.

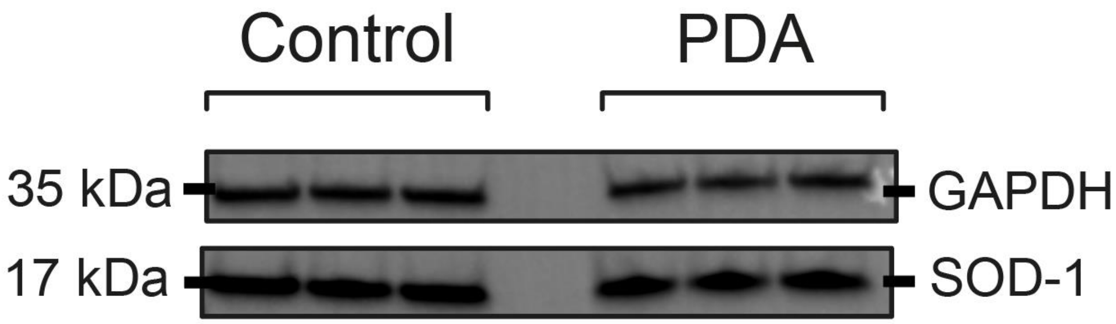

Supplementary Figure S16. Western-blot analysis of SOD-1 and GAPDH proteins in MDA-MB-231 cells treated with $5.0 \cdot 10^{-3}$ mg·mL^{-1} of PDA for 24 h.

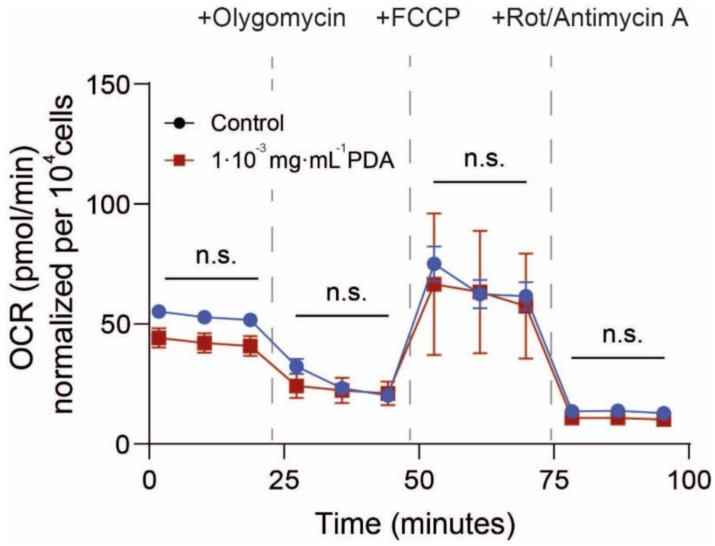

Supplementary Figure S17. OCR analysis in MCF-10A after the treatment with $1 \cdot 10^{-3}$ $mg \cdot mL^{-1}$ PDA for 24 h. The measurement was performed by Seahorse analyzer, by adding 1 µM of oligomycin after 28 min, FCCP (1 µM) after 54 min, and a 1:1 mixture of rotenone A/antimycin A (0.5 µM) after 80 min. ns, not statistically significant. Error bars denote S.E.M (n = 9 per condition).

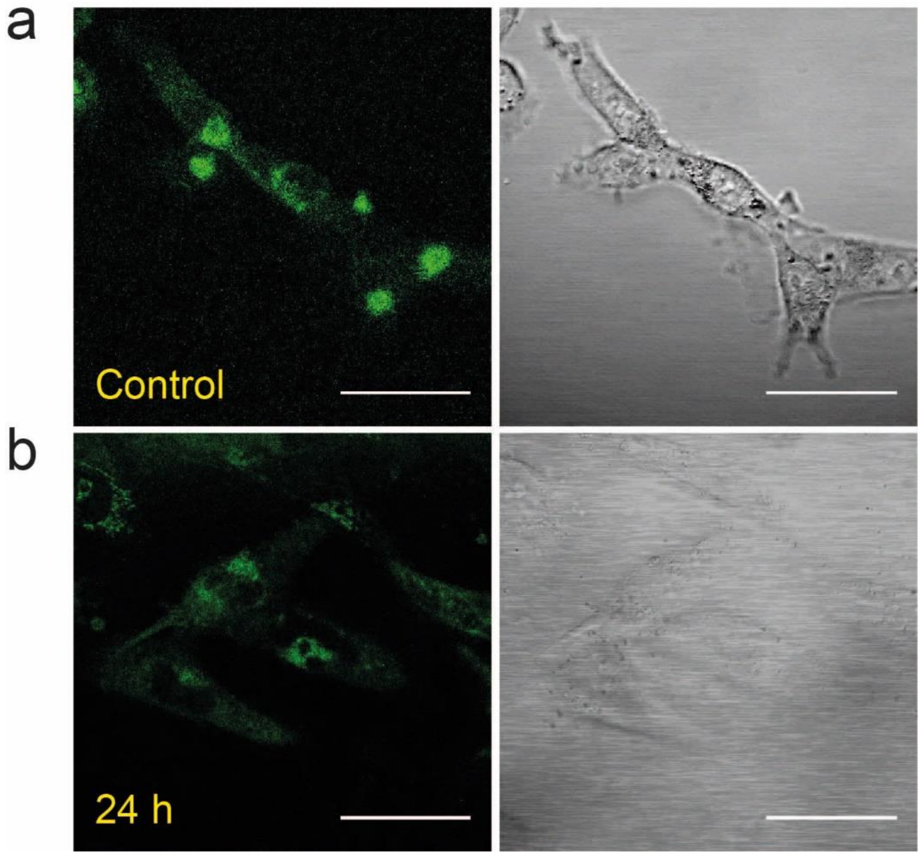

Supplementary Figure S18. Confocal microscopy analysis and bright field images of mitochondrial membrane potential (ΔΨ) imaging JC-1 monomers in (a) control MDA-MB-231 cells and (b) MDA-MB-231 cells incubated with $1.14 \cdot 10^{-3}$ $mg \cdot mL^{-1}$ of PDA for 24 h. Scale bar = 50 μm.

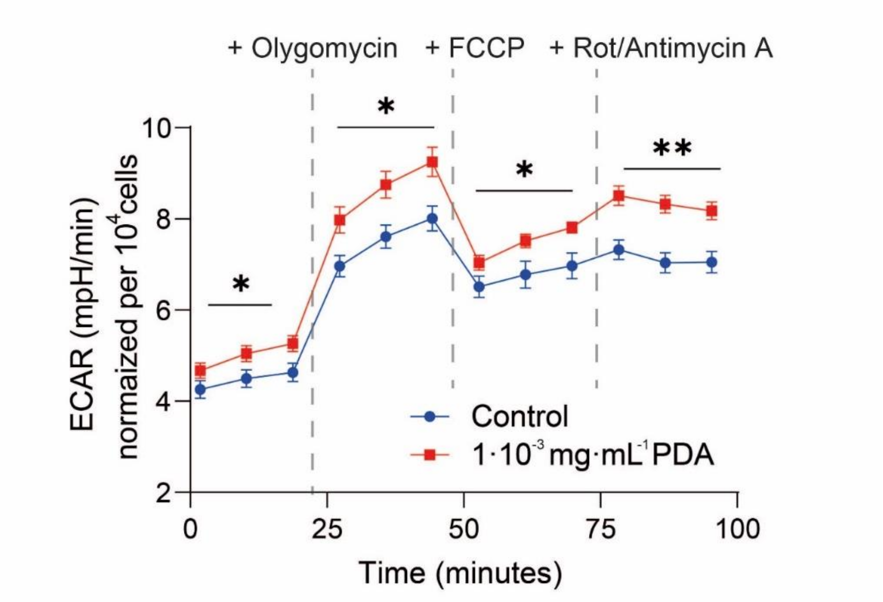

Supplementary Figure S19. Extracellular acidification rate (ECAR) analysis of MDA-MB-231 cells treated with $1.14 \cdot 10^{-3}$ mg·mL^{-1} of PDA nanoparticles for 24 h. The measurement was performed by Seahorse analyzer, by adding 1 µM of oligomycin after 28 min, FCCP (1 µM) after 54 min, and a 1:1 mixture of rotenone A/antimycin A (0.5 µM) after 80 min. Error bars denote S.E.M (n = 9). *P < 0.05, **P < 0.01, ***P < 0.001, and ****P < 0.0001; ns, not statistically significant.

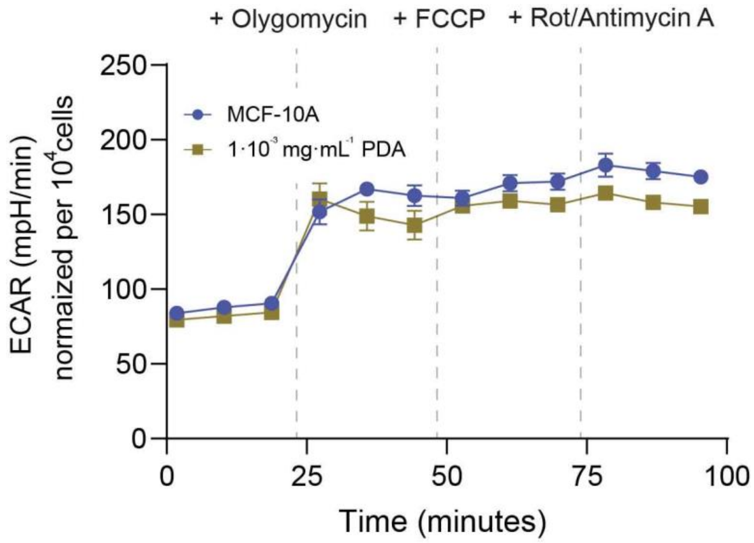

Supplementary Figure S20. ECAR analysis of MCF-10A cells treated with $1.14\cdot10^{-3}$ $mg\cdot mL^{-1}$ of PDA nanoparticles for 24 h. The measurement was performed by Seahorse analyzer, by adding 1 µM of oligomycin after 28 min, FCCP (1 µM) after 54 min, and a 1:1 mixture of rotenone A/antimycin A (0.5 µM) after 80 min. Error bars denote S.E.M (n = 9).

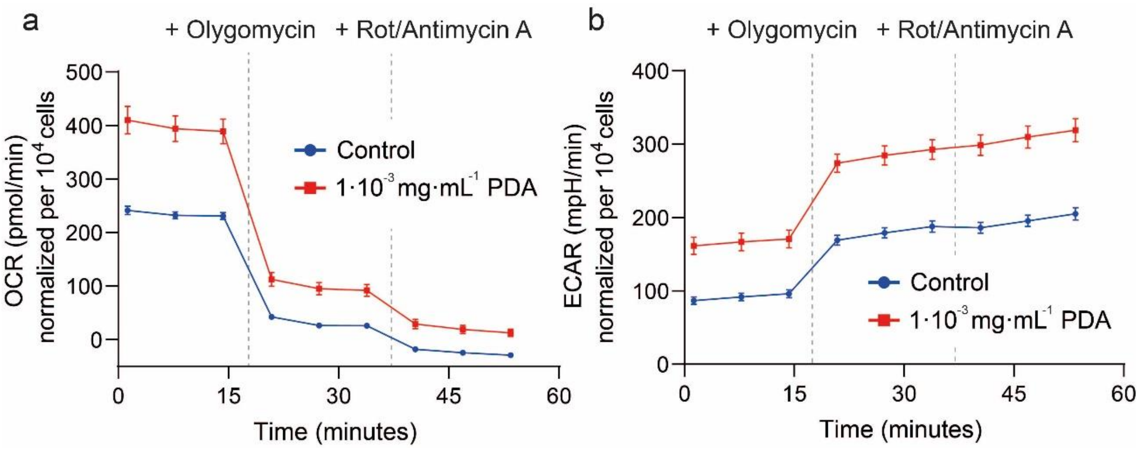

Supplementary Figure S21. (a) OCR and (b) ECAR measurements of MDA-MB-231 cells treated with $1.14 \cdot 10^{-3}$ $mg \cdot mL^{-1}$ of PDA nanoparticles for 24 h. The measurement was performed by Seahorse analyzer, by adding 1 µM of oligomycin after 18 min and a 1:1 mixture of rotenone A/antimycin A (0.5 µM) after 38 min. Error bars denote S.E.M (n = 9).

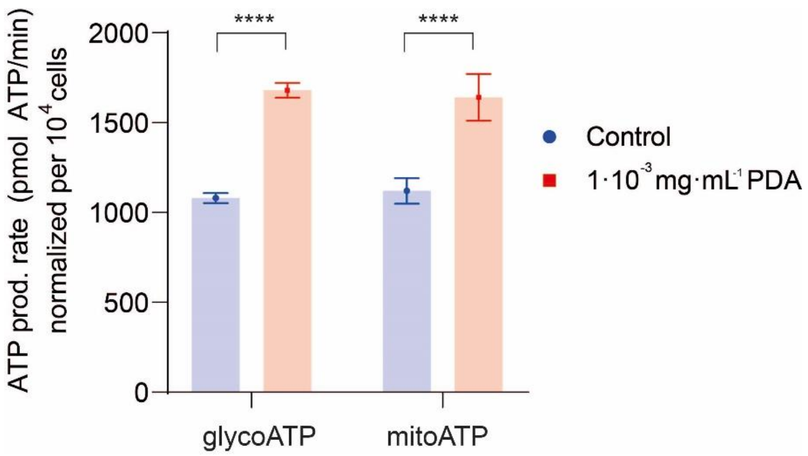

Supplementary Figure S22. Quantification of ATP production rate according to its source whether glycolytic route or oxidative phosphorylation at mitochondria in MDA-MB-231 treated with $1 \cdot 10^{-3}$ mg·mL^{-1} PDA for 24 h. Error bars denote S.E.M (n = 9). *$P < 0.05$, **$P < 0.01$, ***$P < 0.001$, and ****$P < 0.0001$; ns, not statistically significant.

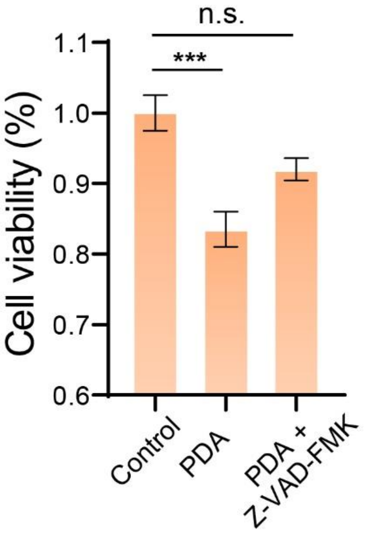

Supplementary Figure S23. MDA-MB-231 viability after 24 h in the presence of $1.14 \cdot 10^{-3}$ $mg \cdot mL^{-1}$ of PDA or $1.14 \cdot 10^{-3}$ $mg \cdot mL^{-1}$ of PDA with 10 µM of Z-VAD-FMK, a well-known apoptosis inhibitor. *P < 0.05, **P < 0.01, ***P < 0.001, and ****P < 0.0001; ns, not statistically significant. Error bars denote S.E.M (n = 3).

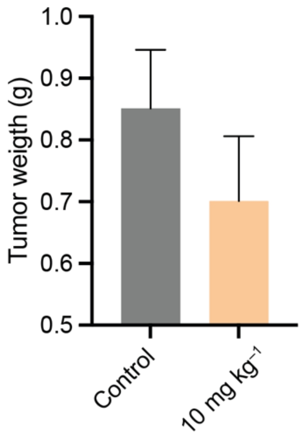

Supplementary Figure S24. Comparison of tumor weight in control and treated group with 10 mg·kg^{-1} of PDA nanoparticles.

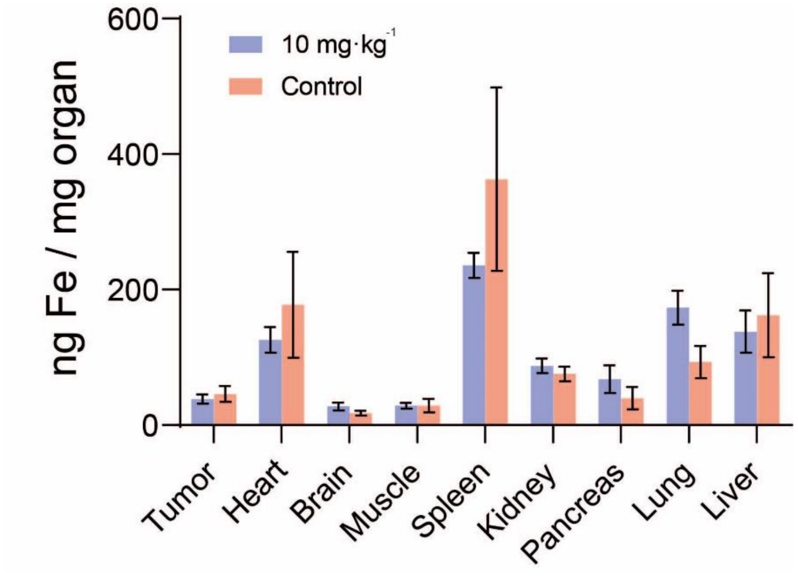

Supplementary Figure S25. Concentration of iron found in different organs for control group (red bar) and 10 mg·kg^{-1} group (blue bar). Error bars denote S.E.M (n=3). Differences between control and treated group were not statistically significant for all organs.

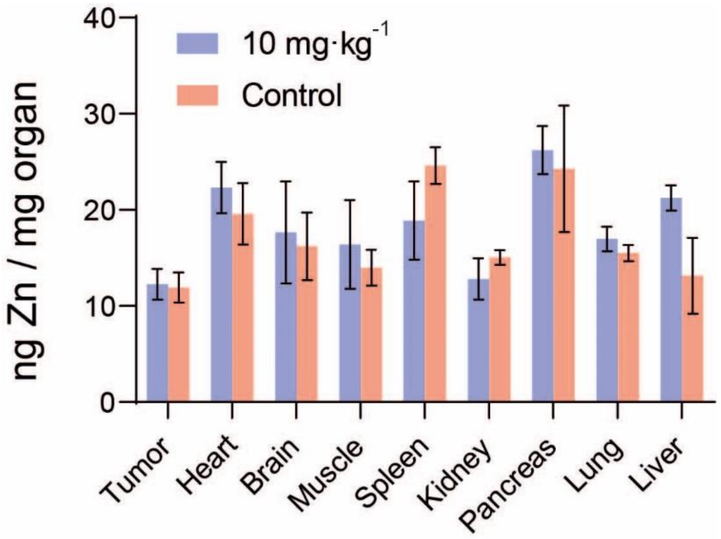

Supplementary Figure S26. Concentration of zinc found in different organs for control group (red bar) and 10 mg·kg^{-1} group (blue bar). Error bars denotes S.E.M (n=3). Differences between control and treated group were not statistically significant for all organs.

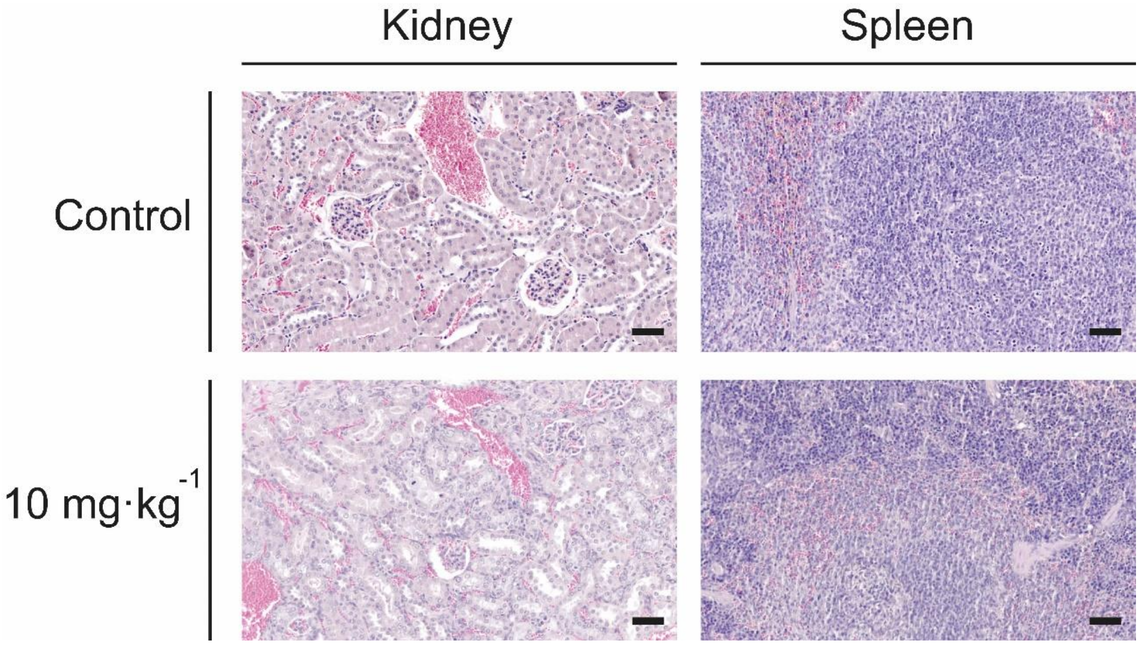

Supplementary Figure S27. Hematoxylin & eosin (H&E)-stained tissue sections from kidney and spleen of tumor-bearing mice in control and 10 mg·kg^{-1} groups. Scale bar: 20 μm.

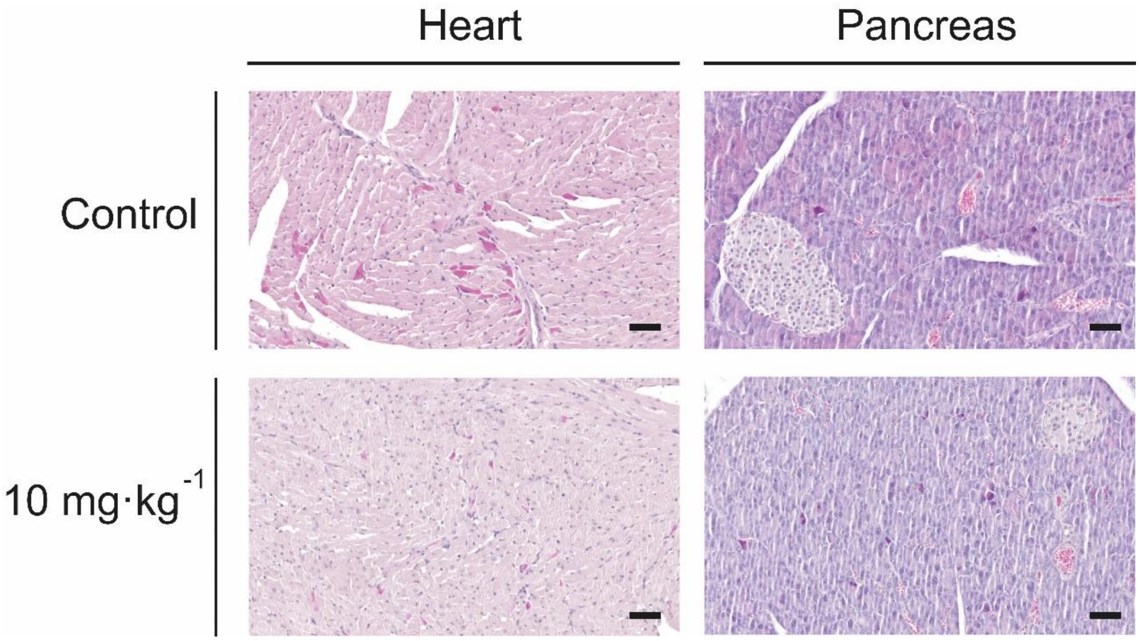

Supplementary Figure S28. H&E-stained tissue sections from heart and pancreas of tumor-bearing mice in control and 10 mg·kg^{-1} groups. Scale bar: 20 μm.

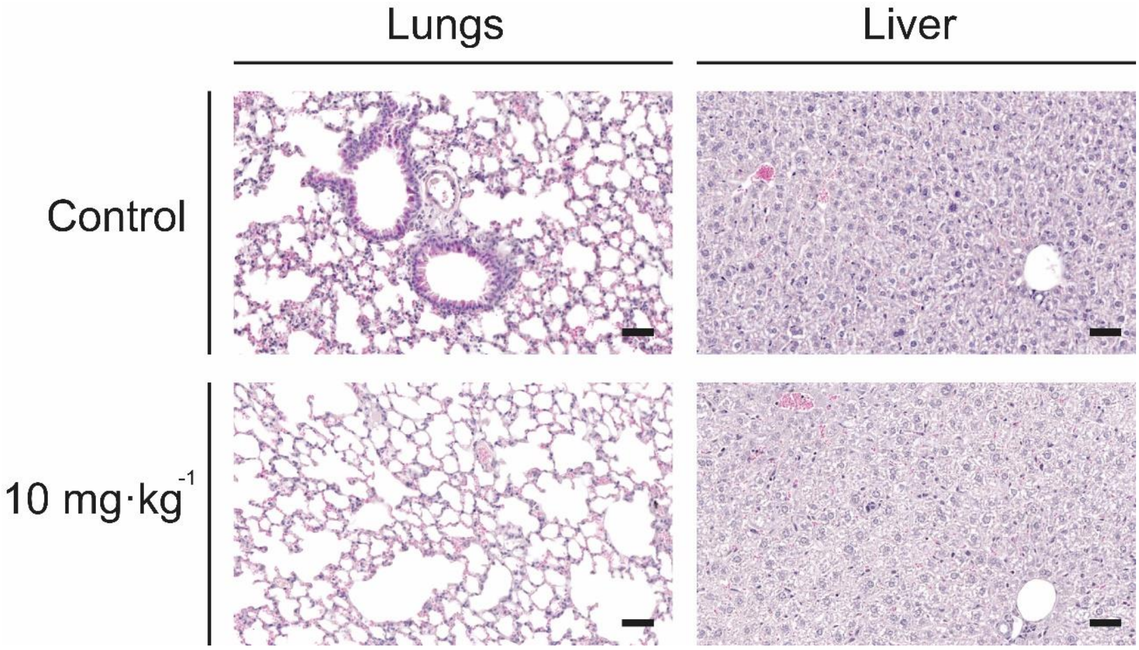

Supplementary Figure S29. H&E-stained tissue sections from lungs and liver of tumor-bearing mice in control and 10 mg·kg^{-1} groups. Scale bar: 20 μm.
